# Activating sulfur oxidation reaction *via* six-electron redox mesocrystal NiS_2_ for sulfur-based aqueous batteries

**DOI:** 10.1093/nsr/nwac268

**Published:** 2022-11-25

**Authors:** Zhoudong Yang, Boya Wang, Yongjin Chen, Wanhai Zhou, Hongpeng Li, Ruizheng Zhao, Xinran Li, Tengsheng Zhang, Fanxing Bu, Zaiwang Zhao, Wei Li, Dongliang Chao, Dongyuan Zhao

**Affiliations:** Laboratory of Advanced Materials, Shanghai Key Laboratory of Molecular Catalysis and Innovative Materials, College of Chemistry and Materials, Fudan University, Shanghai 200433, China; Laboratory of Advanced Materials, Shanghai Key Laboratory of Molecular Catalysis and Innovative Materials, College of Chemistry and Materials, Fudan University, Shanghai 200433, China; Center for High Pressure Science and Technology Advanced Research, Beijing 100094, China; Laboratory of Advanced Materials, Shanghai Key Laboratory of Molecular Catalysis and Innovative Materials, College of Chemistry and Materials, Fudan University, Shanghai 200433, China; Laboratory of Advanced Materials, Shanghai Key Laboratory of Molecular Catalysis and Innovative Materials, College of Chemistry and Materials, Fudan University, Shanghai 200433, China; Laboratory of Advanced Materials, Shanghai Key Laboratory of Molecular Catalysis and Innovative Materials, College of Chemistry and Materials, Fudan University, Shanghai 200433, China; Laboratory of Advanced Materials, Shanghai Key Laboratory of Molecular Catalysis and Innovative Materials, College of Chemistry and Materials, Fudan University, Shanghai 200433, China; Laboratory of Advanced Materials, Shanghai Key Laboratory of Molecular Catalysis and Innovative Materials, College of Chemistry and Materials, Fudan University, Shanghai 200433, China; Laboratory of Advanced Materials, Shanghai Key Laboratory of Molecular Catalysis and Innovative Materials, College of Chemistry and Materials, Fudan University, Shanghai 200433, China; Laboratory of Advanced Materials, Shanghai Key Laboratory of Molecular Catalysis and Innovative Materials, College of Chemistry and Materials, Fudan University, Shanghai 200433, China; Laboratory of Advanced Materials, Shanghai Key Laboratory of Molecular Catalysis and Innovative Materials, College of Chemistry and Materials, Fudan University, Shanghai 200433, China; Laboratory of Advanced Materials, Shanghai Key Laboratory of Molecular Catalysis and Innovative Materials, College of Chemistry and Materials, Fudan University, Shanghai 200433, China; Laboratory of Advanced Materials, Shanghai Key Laboratory of Molecular Catalysis and Innovative Materials, College of Chemistry and Materials, Fudan University, Shanghai 200433, China

**Keywords:** sulfur-based aqueous battery, mesocrystal material, sulfur oxidation reaction, high-energy aqueous battery, six-electron redox

## Abstract

Sulfur-based aqueous batteries (SABs) are deemed promising candidates for safe, low-cost, and high-capacity energy storage. However, despite their high theoretical capacity, achieving high reversible value remains a great challenge due to the thermodynamic and kinetics problems of elemental sulfur. Here, the reversible six-electron redox electrochemistry is constructed by activating the sulfur oxidation reaction (SOR) process of the elaborate mesocrystal NiS_2_ (M-NiS_2_). Through the unique 6e^−^ solid-to-solid conversion mechanism, SOR efficiency can reach an unprecedented degree of *ca.* 96.0%. The SOR efficiency is further revealed to be closely associated with the kinetics feasibility and thermodynamic stability of the M-NiS_2_ intermedium in the formation of elemental sulfur. Benefiting from the boosted SOR, compared with the bulk electrode, the M-NiS_2_ electrode exhibits a high reversible capacity (1258 mAh g^−1^), ultrafast reaction kinetics (932 mAh g^−1^ at 12 A g^−1^), and long-term cyclability (2000 cycles at 20 A g^−1^). As a proof of concept, a new M-NiS_2_‖Zn hybrid aqueous battery exhibits an output voltage of 1.60 V and an energy density of 722.4 Wh kg_cath_^−1^, which opens a new opportunity for the development of high-energy aqueous batteries.

## Teaser

Taking mesocrystal NiS2 as a model sulfur intermedium, we propose and figure out the kinetic and thermodynamic problems of sulfur oxidation reaction (SOR) process in S-based aqueous batteries.

## INTRODUCTION

Aqueous batteries (ABs) have become a hotspot in the last few years due to the merits of high safety, low-cost, non-toxic, and high ion conductivity (∼1 S cm^−1^) [1], which have been regarded as promising candidates in large-scale energy storage systems. However, their intrinsic deficiency in energy density still hardly meets practical demands due to the low output voltage and limited specific capacity (<600 mAh g^−1^) [1–3]. To further promote the energy density of ABs, it is imperative to broaden the electrochemical stable window (ESW) of the aqueous electrolyte and pursue aqueous-compatible electrodes with a high specific capacity [4]. So far, great efforts have been devoted to extending the ESW, including concentrated electrolytes [5], decoupling electrolytes, and modifying the electrolyte/electrode interface [6,7]. In exploring high-capacity aqueous-compatible electrochemistry, sulfur-based aqueous batteries (SABs) with high theoretical capacity hold promise for breaking the bottleneck of energy density [8].

Elemental sulfur features top-level specific capacity (typically >1000 mAh g^−1^) *via* two-electron transfer reactions. In studies of SABs, aqueous metal-sulfur batteries, including Cu-S [9–11], Fe-S [12], Zn-S [13,14], Pb-S [15], Ca-S [16], Li-S [17] and Na-S [18], have attracted much recent attention due to their high actual specific capacity in aqueous electrolytes. Despite some impressive results so far, the electrically insulated elemental sulfur (5 × 10^−30^ S cm^−1^) inevitably undergoes a distinct volume expansion of at least ∼50% during discharging, accompanied by the conversion of dense S_8_ (1.96 g cm^−3^) to M*_x_*S*_y_* (such as Li_2_S, Cu_2_S, and PbS) [19], leading to kinetics and reversibility limitations. The potential H_2_S and O_2_ escape, polysulfides disproportionation, and dissociation doubtlessly degrade the SABs, resulting in the intrinsic loss of active species. Compared with elemental sulfur, transition metal sulfides (TMSs) with high electronic conductivity and tunable structure are regarded as promising aqueous-compatible sulfur-based electrodes. Moreover, M*_x_*S*_y_* shrinks with ion extraction in charging, generating space to relieve volume expansion during discharge, thus alleviating structural damage to the electrode. Taking into account the high electric conductivity (1 × 10^−3^ S cm^−1^) and low solubility of CuS, Li *et al.* shifted from the S electrode to the CuS electrode and developed a Cu-CuS SAB, revealing a rate capability of 497 mAh g^−1^ at a high rate of 7.5 A g^−1^ [20]. Recently, Fu *et al.* proposed a periodically stacked CuS-CTAB superlattice electrode, which shows a capacity of 225.3 mAh g_CuS_^−1^ at 0.1 A g^−1^ and stable cycling performance [21]. It can be found that the reliable output-specific capacities of current TMSs are far from that of elemental sulfur [22,23]. The M*_x_*S*_y_* discharge products with limited reactivity significantly suppress the sulfur oxidation reaction (SOR) during charging [24], resulting in low actual sulfur reversibility. It remains a huge challenge to achieve efficient and stable SOR electrochemistry in aqueous solutions because of its complicated/adverse thermodynamic evolution. To this end, there is an urgent need to realize thermodynamic feasible and stable multiple-electron reaction pathways to solve the SOR issue for the current SABs.

Herein, the activated SOR process is realized by a highly reversible 6e^−^ sulfur redox electrochemistry. A thermodynamically feasible all-solid route of S ↔ NiS_2_ ↔ NiS + Cu_2_S is identified through elaborate mesocrystal NiS_2_ (M-NiS_2_), which validly avoids the H_2_S and O_2_ escape, polysulfides redox shuttling, and the parasitic reactions. SOR efficiency can eventually be promoted *via* kinetics feasibility and thermodynamic stability of M-NiS_2_ intermedium in the formation of elemental sulfur. As a result, the M-NiS_2_ electrode achieves an unprecedented SOR degree of 96.0%, which promotes the overall electrochemical properties, *i.e.* rate capability of 932 mAh g^−1^ at 12 A g^−1^ and long-term rate stability of 2000 cycles at 20 A g^−1^. As a proof-of-concept, the M-NiS_2_‖Zn hybrid aqueous battery exhibits an outstanding specific energy density of 722.4 Wh kg_cath_^−1^ with a low polarization of 0.13 V.

## RESULTS AND DISCUSSION

### Synthesis and characterizations of mesocrystal NiS_2_

**Figure 1.**
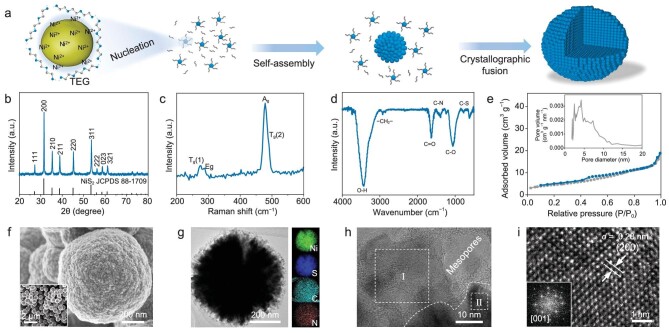
Synthesis and structural characterizations of M-NiS_2_. (a) Schematic illustration of the proposed formation process of M-NiS_2_. (b) XRD pattern. (c) Raman spectrum. (d) FTIR spectrum. (e) N_2_ adsorption-desorption isotherm with pore size distribution as the inset. (f) Enlarged SEM image with a large-scale view as inset. (g) The bright-field TEM image, and corresponding EDX elemental mapping of Ni, S, C, and N in STEM mode. (h and i) Atomic-resolution HAADF-STEM image. The inset is the FFT pattern.

The NiS_2_ nanospheres with mesocrystal structure were synthesized by a facile surfactant-free solvothermal method (Fig. 1a). At 180°C solvothermal conditions, elemental sulfur (with a melting point of ∼115°C) is gradually melted to form spherical droplets in a high-boiling trimethylene glycol (TEG) solvent (boiling point of ∼285°C). During the reaction process, the oxidation products of TEG adsorb on the surface of the primary NiS_2_ particles, enabling the primary NiS_2_ particles to be temporarily stabilized in the solution [25,26]. Subsequently, these primary particles self-assemble to form nanospheres with a selective orientation, ensuring the lowest surface energy [27]. With the gradual prolongation of reaction time, larger NiS_2_ nanospheres are formed (Supplementary Fig. 1). It should be noted that TEG, as a surface stabilizer and reducing agent, plays a significant role in the formation of the M-NiS_2_ [26,28,29].

The crystalline phase of the as-prepared M-NiS_2_ sample can be well-indexed to the cubic NiS_2_ (JCPDS 88-1709, space group: *Pa-3*) with the cell parameters of *a* = *b* = *c* = 5.6873 Å (Fig. 1b). The sharp X-ray diffraction (XRD) peaks indicate the high purity and good crystallinity of the as-prepared M-NiS_2_. The intensities of the reflection peak for (200), (210), (220) and (311) planes slightly deviate from the randomly oriented standard diffraction pattern (Supplementary Fig. 2), suggesting a preferential growth of the M-NiS_2_ [30,31]. The two weak peaks around 275 cm^−1^ in the Raman spectrum can be attributed to the S-S pair vibrations of T_g_(1) and E_g_ modes, while the two strong peaks at about 480 cm^−1^ correspond to stretching modes of the S-S pair (A_g_, T_g_(2)) in the M-NiS_2_ (Fig. 1c) [32]. Furthermore, the characteristic peak at 854.1 (Ni 2p_3/2_) in the Ni 2p high-resolution X-ray photoelectron spectroscopy (XPS) can be ascribed to Ni^2+^ in NiS_2_ (Supplementary Fig. 3a and b) [33,34]. There are two distinct peaks located at 162.8 (S 2p_3/2_) and 164.0 eV (S 2p_1/2_) in the S 2p spectrum (Supplementary Fig. 3c), which are consistent with S_2_^2−^ in NiS_2_ [35]. Besides, the C-S characteristic peaks (164.6 and 166.0 eV) indicate the chemical bond between NiS_2_ and TEG reactant in the M-NiS_2_, which originates from the reaction between TEG and elemental sulfur during the solvothermal process [36]. In addition, the main peaks in the Dual Electron Energy Loss Spectrum (Dual-EELS) profile are in good agreement with the characteristic peaks of Ni^2+^ (Supplementary Fig. 4) [37]. Fourier transform infrared spectroscopy (FTIR) in Fig. 1d confirms the presence of weak C-N [38] and C-S [39] *etc.* vibration peaks, indicating the existence of the TEG reactant in the M-NiS_2_ [40].

The non-tight packing behavior and the meso-channels of M-NiS_2_ sub-nanoparticles are further confirmed by the N_2_ adsorption-desorption isotherms. The isotherms, belonging to the typical IV curves, manifest the presence of mesopores in the M-NiS_2_ with a specific surface area of 16 m^2^ g^−1^ (Fig. 1e). The pore size distribution based on the Barrett-Joyner-Halenda (BJT) model is centered at a range of 2–5 nm. Such favorable morphological features are looking forward to being beneficial for electrolyte infiltration, S reduction/oxidation accommodation, and electrochemical kinetics. The typical scanning electron microscopy (SEM) images of M-NiS_2_ show a homogeneous nanosphere morphology (500–700 nm) piled up by nanoparticles (Fig. 1f and Supplementary Fig. 5). The transmission electron microscopy (TEM) image further depicts that the M-NiS_2_ nanospheres consist of numerous primary particles (Supplementary Fig. 6), which is well consistent with the morphology of the SEM results. Energy Dispersive X-ray (EDX) mapping further confirms the uniform distribution of S and Ni (the molar ratio of Ni and S is 0.499), while the presence of C and N can be ascribed to the TEG reactant (Fig. 1g and Supplementary Fig. 7), corresponding to FTIR results.

The high-angle annular dark-field scanning transmission electron microscopy (HAADF-STEM) result illustrates that the M-NiS_2_ nanosphere is crystallographically coaligned (Fig. 1h and enlarged detail information in Supplementary Fig. 8). This is confirmed by the corresponding Fast Fourier Transform (FFT) patterns taken from regions I and II, which exhibit the same orientation and can be indexed as the *Pā3* phase along the [001] zone axis (see Supplementary Fig. 9a–c). The FFT patterns of the whole region further illustrate that the primary nanoparticles are of the same [001] zone axis with an orientation difference of 45° (Supplementary Fig. 9d and e) [41]. Furthermore, the lattice spacing of 0.28 nm in the atomic-resolution HAADF-STEM image belongs to the (200) crystal plane of M-NiS_2_ along the [001] zone axis (Fig. 1i). The above results confirm that the synthesized NiS_2_ nanoparticles are of mesocrystal and mesopore features.

### Electrochemical behavior

**Figure 2.**
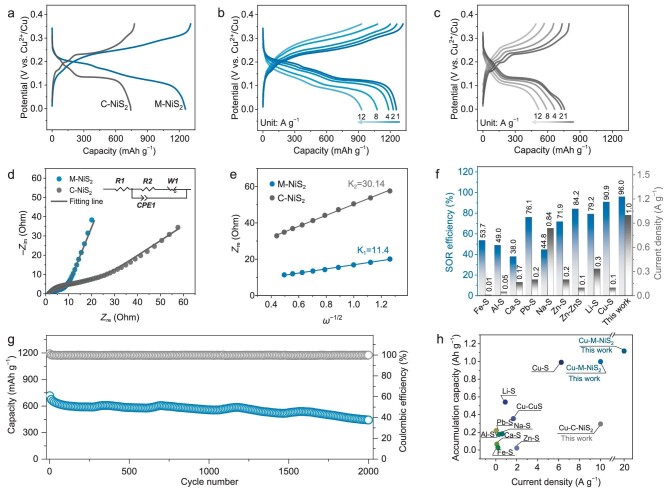
Electrochemical performances of M-NiS_2_ electrode. (a) GCD plots and rate capability of the (b) M-NiS_2_ and (c) C-NiS_2_ electrodes after activation. The capacities are based on the active mass of NiS_2_. (d) Initial EIS spectra of the M-NiS_2_ and C-NiS_2_ electrodes at fresh state. An inset equivalent circuit of *R_1_(CPE_1_(R_2_W_1_))* is used to simulate the resistances, where *R_1_, R_2_, CPE_1_*, and *W_1_* are the ohmic resistance of solution and electrodes, the charge-transfer resistance, the double-layer capacitance, and the Warburg impedance, respectively. (e) The relationship between *Z*_re_ and *ω*^−1/2^ in the low-frequency region. (f) SOR efficiency of various SABs. (g) Long-term cycle performance of the M-NiS_2_ electrode at 20 A g^−1^. (h) Cumulative capacity for different SABs. The accumulation capacity can be obtained by accumulating the cycle capacities at the corresponding current density.

The electrochemical performances of the M-NiS_2_ nanospheres were evaluated in comparison with the commercial NiS_2_ (C-NiS_2_) (9). The M-NiS_2_ electrode affords a high reversible specific capacity of 1258 mAh g^−1^ at 1 A g^−1^ after 10 cycles of activation (Supplementary Fig. 11) based on the mass of M-NiS_2_, which is close to the theoretical value of the six-electron transfer reaction of NiS_2_ (Fig. 2a). Note that almost all of the actual capacity comes from M-NiS_2_, as the carbon cloth, slurry ingredients, and H^+^ ions are inactive in the electrolyte (Supplementary Fig. 12). In addition, the M-NiS_2_ exhibits a high specific capacity of 932 mAh g^−1^ when the current density reaches 12 A g^−1^ (Fig. 2b). In contrast, the specific capacity of the C-NiS_2_ electrode is only 755 mAh g^−1^ at 1 A g^−1^ and 516 mAh g^−1^ at 12 A g^−1^, indicating its inadequate electrochemical activity (Fig. 2c). Moreover, the rate cycling results show that the M-NiS_2_ electrode promises robust rate performance (Supplementary Fig. 13).

The excellent electrochemical performances of the M-NiS_2_ mesocrystal are associated with its high electrical conductivity and ionic transportation. The electronic conductivity of the M-NiS_2_ and C-NiS_2_ are 15.53 and 0.17 S cm^−1^, respectively, indicating a faster electron transport network of the M-NiS_2_ (Supplementary Table 1). The existence of mesopores within the M-NiS_2_ material is conducive to electrolyte penetration and ionic transportation, which was certified by electrochemical impedance spectroscopy (EIS). Compared with C-NiS_2_, the M-NiS_2_ electrode exhibits lower charge transfer resistance of 4.0 Ω (*vs.* 16.6 Ω for C-NiS_2_) EIS (Fig. 2d). The apparent ionic diffusion coefficient (D) can then be calculated based on the relationship between real component resistance (*Z*_re_) and the square root of frequency (*ω*^−1/2^) in the low-frequency region [42]. As shown in Fig. 2e, the D values of the M-NiS_2_ and C-NiS_2_ electrodes were calculated to be 3.3 × 10^−18^ and 4.8 × 10^−19^ cm^2^ s^−1^, respectively, demonstrating facilitated ionic transportation due to the unique mesocrystal structure of the M-NiS_2_ (see supporting information for the calculation).

Apart from the high specific capacity, the M-NiS_2_ also exhibits a high SOR efficiency (defined as a ratio of the reversible charge capacity and the theoretical capacity) of *ca.* 96.0% at 1 A g^−1^. To the best of our knowledge, this value is among the highest of the reported SABs (Fig. 2f) [9,12,14–18,22,43]. Furthermore, the M-NiS_2_ holds high-rates of stability upon long-term cycles. At the moderate current density of 1 and 5 A g^−1^, the capacity retention of the electrode was 96.5% after 200 cycles and 90.9% after 500 cycles, respectively (Supplementary Fig. 14a and b). At a higher current density of 10 A g^−1^, the specific capacity decays from 1059 to 955 mAh g^−1^ after 800 cycles with a capacity retention of 90.1% (Supplementary Fig. 14c; C-NiS_2_ as the comparison can be obtained from Supplementary Fig. 15). Moreover, the M-NiS_2_ electrode demonstrates a long cycling life over 2000 cycles at an ultra-high rate of 20 A g^−1^ with a capacity retention of 61.7% (Fig. 2g). Notably, such remarkable cumulative capacity performance of our M-NiS_2_ under high current densities outperforms most reported SABs (Fig. 2h) [9,12,15–19,43,44]. More importantly, the M-NiS_2_ is also suitable for high-loading electrodes. It still exhibits a high reversible capacity of 1116 mAh g^−1^ at a high loading of 5–7 mg cm^−2^ (Supplementary Fig. 16), which is critical for practical applications.

### S-based electrochemistry and charge storage mechanism

**Figure 3.**
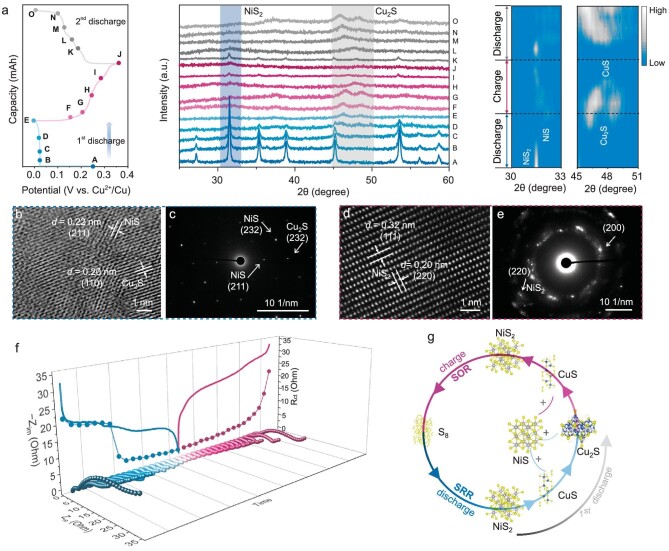
S electrochemistry and charge storage mechanism of M-NiS_2_. (a) GCD profile of the M-NiS_2_ electrode and *ex-situ* XRD patterns at different stages. (b) HAADF-STEM after fully discharging. (c) SAED pattern after fully discharging. (d) HAADF-STEM after fully charging. (e) SAED pattern after fully charging. (f) *In-situ* EIS spectrum and variation of transfer resistance of the M-NiS_2_-Cu cell. (g) A schematic illustration of phase conversion during the cycling process.

As can be seen from *ex-situ* XRD results (Fig. 3a), the intensities of the characteristic peaks of M-NiS_2_ mesocrystals gradually weaken from Point A (P_A_) to Point C (P_C_) in the initial discharge process. They disappear completely in the following discharge process of P_C_ → P_E_, whilst the new diffraction peaks of NiS (JCPDS 86-2281) and Cu_2_S (JCPDS 26-1116) appear, indicating the reduction from S_2_^2−^ to S^2−^ (see more evidence in Supplementary Fig. S17). In the initial charge process of P_E_ → P_H_, the Cu_2_S gradually transforms to CuS (JCPDS 03-1090, as shown in Fig. 3a and 9). Subsequently, the peaks of NiS_2_ appear during the charging process of P_H_ → P_I_, and the formed CuS gradually disappears. Significantly, the peak intensity of NiS_2_ gets weak during the end-of-charge period of P_I_ → P_J_, which insinuates that the regenerated NiS_2_ may undergo further oxidation at this potential, *i.e.* S_2_^2−^ is possible to be oxidized to elemental sulfur at a fully charged state (see more characterizations at the below SOR analysis part). In the second discharge process of P_J_ → P_K_, the diffraction peaks of NiS_2_ in Fig. 3a get stronger again, revealing that the elemental sulfur is first reduced to S_2_^2−^*via* reaction in the vicinity of Ni ions. In the following discharge stage of P_K_ → P_M_, the NiS_2_ phase disappears, accompanied by the emersion of NiS and CuS. Then, the CuS is further reduced to the Cu_2_S with the discharge process proceeding from P_M_ → P_O_, which repeats the progress in the first discharge cycle. The potential of the aforementioned electrochemical phase transition processes also corresponds to the redox peak positions in the cyclic voltammetry (CV) cycle (Supplementary Fig. 18a). Supplementary Fig. 18f further illustrates the phase change involved in the reaction process.

The Ni 2p spectra in Supplementary Fig. 19 shows that the peaks shift from the original NiS_2_ to NiS after the first discharge [45]. After the first charge, the characteristic peaks of Ni almost disappear, which is consistent with the aforementioned XRD result. The SOR process of NiS_2_ can also be reflected by the release of Ni ions into the electrolyte at the first fully charged state (see relevant Inductive Coupled Plasma Emission Spectrometer analysis in Supplementary Table 2). No Ni ions can be detected in the electrolyte after the first discharge, indicating that Ni exists in the form of solid NiS in the process of the first sulfur reduction reaction (SRR). Similarly, after the second discharge, neglectable Ni ions can be detected in the solution, indicating the updates of Ni^2+^ in the second SRR process, and the Ni restores in the electrode in the form of a solid.

Furthermore, the HAADF-STEM investigations permit direct visualization of the aforementioned solid-to-solid phase conversion processes. At the fully discharged state, the NiS phase with the (211) plane and Cu_2_S phase with the (110) plane can be observed (Fig. 3b), which are also confirmed by the selected area electron diffraction (SAED) result (Fig. 3c). Moreover, the simultaneous detection of EDX and EELS data accurately indicate the separation of NiS and Cu_2_S phases (9). After full charging, only a slight amount of NiS_2_ crystalline phase remains, which can be revealed by the HAADF-STEM and SAED results (Fig. 3d and e, and Supplementary Fig. 21). Herein, the residual NiS_2_ can form a conductive network for elemental sulfur accommodation and acceleration of the electron transport.

The reversible solid-to-solid phase conversion change after activation can also be reflected by the *in-situ* EIS test. The charge transfer resistance (*R_ct_*) value decreases from 21.6 to 2.1 Ω during discharge, indicating the gradual transformation of the insulating elemental sulfur into high electronic conductivity metal sulfides (Fig. 3f). Then, the impedance presents an increasing trend with the metal sulfides oxidizing to elemental sulfur during charging, and the value gradually increases from 2.1 to 23.1 Ω. It is worth noting that there is a sudden resistance change during the discharge/charge process, which should correspond to the SRR/SOR transition of elemental sulfur. Moreover, the negligible resistance values during the transition between metal sulfides indirectly prove the high electronic conductivity characteristics of the aforementioned metal sulfides. The reversible trend of the *in-situ* EIS spectra also well demonstrates the excellent reversibility of sulfur reduction/oxidation reactions.

We can therefore propose a new 6e^−^ S-based electrochemistry pathway based on the reversible solid-to-solid S ↔ S_2_^2−^ ↔ S^2−^ cascade redox pairs with M-NiS_2_ as the conversation intermedium (Fig. 3g). With the electrochemical reaction between the initial active materials of M-NiS_2_ and the Cu^2+^ charge carrier, the initial SRR undergoes a four-electron reaction of NiS_2_ to NiS and Cu_2_S. During SOR, Cu_2_S and NiS are reversibly oxidized to the NiS_2_ phase and release Cu^2+^ ions concomitantly. It should be noted that, in the charge/discharge process, CuS serves as a transition phase for lowering the reaction barrier for Cu_2_S reduction [9]. Moreover, due to the high contact interface of NiS_2_/electrolyte formed by the mesocrystal structure, the electrochemical reactivity of the M-NiS_2_ electrode can be effectively improved, thus resulting in a facilitated sulfur oxidation reaction. In the subsequent charging process, most NiS_2_ intermedium is further oxidized to elemental sulfur, leaving a slight amount of NiS_2_ residual as a conductive S host (see below discussion). Therefore, a 6e^−^ S-based redox electrochemistry can be constructed *via* the M-NiS_2_ intermedium (6e^−^ comes from the cascade reaction of S/S^2−^ and Cu^2+^/Cu^+^), and the electrochemical reaction pathway can be briefly described as S ↔ NiS_2_ ↔ NiS + Cu_2_S. The consecutive reactions can be formulized as follows (after the initial discharge-charge process):


(1) \begin{eqnarray*}\!\!\!\!\!\!\!\!\!\!\!\!\!\!\!\!\!\!\!\!\!\!\!\!\!\!\!\!\!\!\!\!\!\!\!\!\!{\rm{Step1{:}\ 2S + N}}{{\rm{i}}}^{{\rm{2 + }}}+{\rm{ 2}}{{\rm{e}}}^{\rm{ - }} \leftrightarrow {\rm{Ni}}{{\rm{S}}}_{\rm{2}}\end{eqnarray*}

(2) \begin{eqnarray*}\!\!\!\!{\rm{Step2{:}\ Ni}}{{\rm{S}}}_{\rm{2}}\,{\rm{ + C}}{{\rm{u}}}^{{\rm{2 + }}}+{\rm{ 2}}{{\rm{e}}}^{\rm{ - }} \leftrightarrow {\rm{NiS + CuS}}\end{eqnarray*}

(3) \begin{eqnarray*}\!\!\!\!\!\!\!\!\!\!\!\!\!\!\!\!\!{\rm{Step3{:}\ CuS + C}}{{\rm{u}}}^{{\rm{2 + }}}+{\rm{ 2}}{{\rm{e}}}^{\rm{ - }} \leftrightarrow {\rm{C}}{{\rm{u}}}_{\rm{2}}{\rm{S}}\end{eqnarray*}

### SOR kinetics and thermodynamics analyses

**Figure 4.**
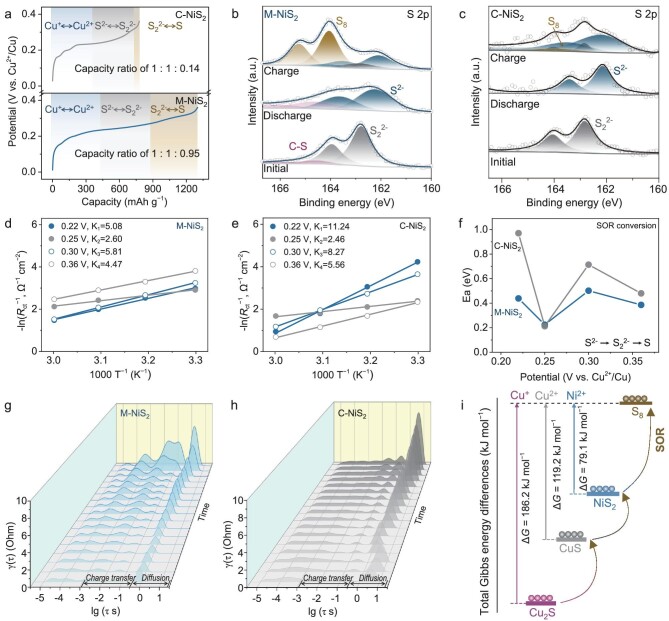
SOR kinetics and thermodynamics analyses. (a) SOR performance of M-NiS_2_ and C-NiS_2_ electrodes at 1 A g^−1^. XPS spectra of S 2p for (b) M-NiS_2_ and (c) C-NiS_2_ at different charge/discharge stages; Arrhenius-plots of *R_ct_* values for (d) M-NiS_2_ and (e) C-NiS_2_. (f) The activation energy for the M-NiS_2_ and C-NiS_2_ electrodes at varying voltage. DRT plot of *in-situ* EIS data during charging for (g) M-NiS_2_-Cu cell and (h) C-NiS_2_-Cu cell. (i) The Gibbs energy changes of different conversation intermedium in the SOR process.

The SOR processes of both M-NiS_2_ and C-NiS_2_ electrodes are further compared by the galvanostatic charge-discharge (GCD) (Fig. 4a). Obviously, it can be found that the M-NiS_2_ electrode can be charged to a much higher capacity compared with the C-NiS_2_ one, suggesting a higher sulfur oxidation degree. The capacity contribution ratios of (Cu^+^ → Cu^2+^) : (S^2−^ → S_2_^2−^) : (S_2_^2−^ → S) can be roughly estimated as 1 : 1 : 0.14 for C-NiS_2_ and 1 : 1 : 0.95 for M-NiS_2_, respectively. Collaboratively, XPS spectra of S 2p results show two new main intensity peaks at 164.0 and 165.2 eV for the M-NiS_2_ electrode after charging (Fig. 4b), corresponding to S 2p_3/2_ and S 2p_1/2_ of elemental sulfur, respectively [46]. In contrast, the charge product of the C-NiS_2_ electrode remains the main species NiS_2_, with extremely weak characteristic peaks from elemental sulfur (Fig. 4c). The result is further confirmed by *ex-situ* XRD (Supplementary Fig. 22).

Interestingly, the appearance of elemental sulfur indicates that M-NiS_2_ as a transition metal sulfide may have the effect of catalyzing the kinetics of sulfur-polysulfides conversion. To reveal the potential SOR catalysis effect, electrochemical kinetics were investigated *via* the determination of the energy barrier (*E_a_*) [47,48]. The EIS curves of electrodes at 0.22, 0.25, 0.30 and 0.36 V during different SOR processes were measured at a series of temperatures of 303, 313, 323 and 333 K, respectively (Supplementary Figs 23 and 24). *E_a_* at each voltage can then be obtained by fitting the *R_ct_* values using the Arrhenius equation (Fig. 4d and e, and Supplementary Table 3). As shown in Fig. 4f, the *E_a_* for the M-NiS_2_ electrode at 0.22 V is 0.44 eV, less than that for the C-NiS_2_. For the SOR processes at 0.30 and 0.36 V (S_2_^2−^ → S), the *E_a_* values for the M-NiS_2_ are 0.50 and 0.39 eV, respectively, which are also lower than that of the C-NiS_2_. In addition, the redox kinetics of electrode materials was also investigated by the CV technique (from 0.04 to 0.20 mV s^−1^) according to the Randles–Sevcik equation (Supplementary Fig. 25) [49,50]. The M-NiS_2_ exhibits enhanced overall electrochemical kinetics during the SOR process compared with the C-NiS_2_. These results indicate that the M-NiS_2_ electrode exhibits a potential catalysis effect on the sulfur-polysulfides redox reactions, which should be explored in our following studies.

The SOR kinetics were further investigated by *in-situ* EIS measurements in charging mode. The distribution of relaxation time (DRT) analysis was utilized to identify relaxation processes during charging [51,52]. For the M-NiS_2_ electrode (Fig. 4g), the relaxation interval of 0.35–6.54 s corresponds to the ion diffusion process, and the polarization resistance associated with the diffusion process increases from 1.66 to 6.54 Ω. It is worth noting that at the end of SOR, the bimodal polarization resistance in the interval corresponding to the charge transfer relaxation shows a sharp increase (3.65 ms–0.44 s) from 0.83 and 0.42 to 4.11 and 3.94 Ω, respectively. This phenomenon may be associated with the formation of elemental sulfur with low electronic conductivity. In contrast, the overall diffusion kinetics of the C-NiS_2_ electrode is relatively hysteretic (Fig. 4h), with a diffusion relaxation interval of 0.58–15.85 s, and the diffusion polarization resistance increases from 1.47 to 8.57 Ω. Significantly, at the end of SOR in the C-NiS_2_, neglectable polarization resistances in the charge transfer relaxation region can be observed. The above results further illustrate that the M-NiS_2_ with mesocrystal and mesopore features performs not only better ionic diffusion but also higher reaction kinetics, which is conducive to the SOR process for elemental sulfur formation.

In terms of thermodynamics, the achievement of high SOR efficiency and the role of NiS_2_ can be further understood based on the Gibbs free energy calculation (Fig. 4i). The change in Gibbs free energy is 79.1 kJ mol^−1^ for NiS_2_ → S, which is much lower than that for the routes of Cu_2_S → S (186.2 kJ mol^−1^) and CuS → S (119.2 kJ mol^−1^), indicating the feasible thermodynamics advantage for NiS_2_ as a conversation intermedium being oxidized to elemental sulfur. Hence, the SOR process can be feasibly achieved by stepwise solid-to-solid phase conversation of S^2−^ → S_2_^2−^ → S.

## Full cell validation of M-N iS_2_**‖**Z n hybrid aqueous battery

**Figure 5.**
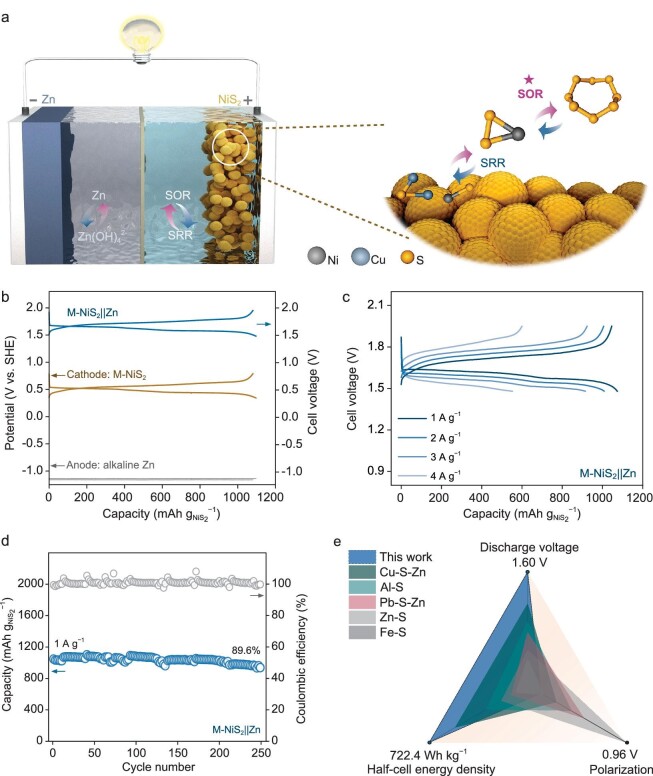
Full device validation of M-NiS_2_‖Zn hybrid aqueous battery. (a) Schematic illustration of the M-NiS_2_‖Zn hybrid aqueous battery. (b) GCD potential curves of the M-NiS_2_ cathode, the alkaline Zn anode, and the hybrid full cell. (c) GCD profiles at varied current densities. (d) Cycling stability. (e) Comparison of discharge voltages, polarization voltages, and energy density for different SABs. The applied current densities are 1 A g^−1^ for this work, 0.05 A g^−1^ for Cu-S-Zn, 0.05 A g^−1^ for Al-S, 0.5 A g^−1^ for Pb-S-Zn, 0.1 A g^−1^ for Zn-S, and 0.05 A g^−1^ for Fe-S batteries.

The excellent capability of M-NiS_2_ in half cells encourages us to fabricate a device of M-NiS_2_‖Zn hybrid aqueous battery to boost the energy density of SABs (Fig. 5a). Coupling the redox of NiS_2_ (*ca.* 0.46 V *vs.* SHE) with Zn/Zn(OH)_4_^2−^ (*ca.* −1.14 V *vs.* SHE) (Fig. 5b), the output voltage of the M-NiS_2_‖Zn hybrid cell can reach 1.60 V. The electrochemical reactions can then be formulated as follows:


(4) \begin{eqnarray*} \!\!\!\!\!\!\!\!\!\!\!\!\!\!\!\!\!\!\!\!\!\!\!\!\!\!\!{\rm Cathode}{:}\ 2{\rm S} &+& {\rm N}{{\rm{i}}}^{{\rm{2 + }}}+{\rm{ 2C}}{{\rm{u}}}^{{\rm{2 + }}}\\ &&+\,{\rm{ 6}}{{\rm{e}}}^{\rm{ - }} \leftrightarrow {\rm{NiS + C}}{{\rm{u}}}_{\rm{2}}{\rm{S}} \end{eqnarray*}

(5) \begin{eqnarray*} \!\!\!{\rm{Anode{:}\ Zn + 4O}}{{\rm{H}}}^{\rm{ - }} \leftrightarrow {\rm{Zn}}{\left( {{\rm{OH}}} \right)}_{\rm{4}}^{{\rm{2 - }}}+{\rm{ 2}}{{\rm{e}}}^{\rm{ - }} \end{eqnarray*}

The M-NiS_2_‖Zn hybrid aqueous battery displays a prominent electrochemical performance with discharge capacities of 1073, 1008, 915, and 553 mAh g^−1^ at 1, 2, 3, and 4 A g^−1^, respectively (Fig. 5c and Supplementary Fig. 26). Additionally, the hybrid aqueous battery also realizes satisfactory capacity retention of *ca.* 89.6% after 250 consecutive cycles at 1 A g^−1^ (Fig. 5d). In addition, the 140-hours standing experimental result shows that the full battery has a low self-discharge and stable charge-storage ability (Supplementary Fig. 27). Compared with other SABs reported previously (Fig. 5e) [9,12,13,15,43], the designed hybrid aqueous battery not only exhibits a higher output voltage of 1.60 V but also remains a lower polarization voltage of 0.13 V. As a result, the energy density can reach 722.4 Wh kg_cath_^−1^ at 1 A g^−1^ based on the total material mass of NiS/Cu_2_S/KB (see supporting information for the calculation), which outperforms many previously reported SABs (with the same calculation method in Supplementary Table 4). More realistically, the theoretical energy density of the hybrid aqueous battery can be calculated as *ca.* 379.2 Wh kg^−1^, based on the mass from both cathode, anode (N/P ratio of 1 : 1), and just-enough solute for the reaction. Note, the actual energy density of this M-NiS_2_‖Zn hybrid aqueous battery cannot be comparable to state-of-the-art LIBs but may be superior to most other ABs.

## CONCLUSION

In summary, we constructed a highly reversible six-electron redox electrochemistry by activating SOR *via* the elaborated design of M-NiS_2_. A unique solid-to-solid conversion reaction mechanism of S ↔ NiS_2_ ↔ Cu_2_S + NiS has been clearly revealed, which can avoid the redox shuttling and parasitic reaction of polysulfides, favoring the reversibility and stability of SABs. The kinetics feasibility and thermodynamic stability of the M-NiS_2_ intermedium in the formation of elemental sulfur are further revealed to be crucial for achieving high SOR efficiency. The existence of the unique mesocrystal and mesopore features endows the NiS_2_ phase with high ionic diffusion and charge transfer kinetics, resulting in favorable SOR reactivity. As a result, the M-NiS_2_ electrode achieves a high SOR efficiency of *ca.* 96.0%, excellent rate capability (932 mAh g^−1^ at 12 A g^−1^), and long-term rate cyclability (2000 cycles at 20 A g^−1^). By matching with an alkaline Zn anode, the constructed M-NiS_2_‖Zn hybrid aqueous battery delivers a half-cell energy density of 722.4 Wh kg_cath_^−1^. Moreover, the elaborate design with the activated SOR process may advance the theoretical development of current SABs, open a new opportunity for the development of aqueous batteries, and should be of immediate benefit for low-cost practical energy storage and grid-scale applications.

## Supplementary Material

nwac268_Supplemental_File
