# Supplementary material for: Activating sulfur oxidation reaction via six-electron redox mesocrystal NiS2 for sulfur-based aqueous batteries
Source: Natl Sci Rev. 2022 Nov 25;10(6):nwac268. doi: 10.1093/nsr/nwac268 (PMC10171633; doi:10.1093/nsr/nwac268)
Supplement: nwac268_Supplemental_File [file nwac268_supplemental_file.pdf]

Supporting Information

Activating sulfur oxidation reaction *via* six-electron-redox mesocrystal NiS_2 for sulfur-based aqueous battery

Zhoudong Yang[1], Boya Wang[1], Yongjin Chen[2], Wanhai Zhou[1], Hongpeng Li[1], Ruizheng Zhao[1], Xinran Li[1], Tengsheng Zhang[1], Fanxing Bu[1], Zaiwang Zhao[1], Wei Li[1], Dongliang Chao[1,*] and Dongyuan Zhao[1,*]

[1]Laboratory of Advanced Materials, Shanghai Key Laboratory of Molecular Catalysis and Innovative Materials, College of Chemistry and Materials, Fudan University, Shanghai 200433, P. R. China.

[2]Center for High Pressure Science and Technology Advanced Research, Beijing, 100094, China

***Corresponding authors**. E-mails: dyzhao@fudan.edu.cn; chaod@fudan.edu.cn

MATERIALS AND METHODS

Materials

Triethylene glycol (TEG) (>99.0%, GC) and elemental sulfur powder (AR, >99.5%) were purchased from Aladdin. Nickel(II) nitrate hexahydrate ($Ni(NO_3)_2 \cdot 6H_2O$) (AR, >98.0%) was obtained from Sinopharm Chemical Reagent Co. LTD. Commercial NiS_2 (C-NiS_2) was purchased from Bidepharm. The above chemicals were directly used in experiments without any purification treatment.

Characterization and Measurements

The crystallographic structures of the samples were characterized by the Bruker D8 powder X-ray diffractometer (XRD, Germany) with Cu Kα irradiation (λ=1.5406 Å). The scanning electron microscopy (SEM) images were observed by Zeiss GeminSEM500 (Germany). The transmission electron microscopy (TEM), selected area electron diffraction (SAED), and scanning transmission electron microscopy (STEM) images in this work were performed at 200 kV on a probe-corrected ARM 200F microscope (cold field gun @ JEOL, JAPAN) with an energy dispersive X-ray system (200 mm^2, total solid angle 1.75 sr). The high-angle annular dark-field (HAADF) images were acquired with an ADF detector (annular ranges of 54-220 mrad), dwell times per pixel of 16 μ s for 1 k × 1 k image size and 10 μ s for 2 k × 2 k image size were used. To minimize possible specimen damage from electron beam irradiation, the beam current was limited below 40 pA. DualEELS spectra (@ Gatan GIF spectrometer) were recorded by a scanning mode, and the data was processed by energy alignment using the zero-loss peak, together with noise reduction using principal component analysis. The X-ray photoelectron spectra were obtained by Thermo Fischer ESCALAB 250Xi (America) with Mono Al source ($h\nu$ = 1486.6 eV) under vacuum condition of 8×10^{-10} Pa. Raman spectra test was performed on a Dilor LabRam-1B microscopic Raman spectrometer (France) by applying a He-Ne laser with 632.8 nm. Fourier-transform infrared (FTIR) spectra were obtained through the PerkinElmer Spectrum II FTIR Spectrometer. The nitrogen adsorption-desorption isotherm was tested by using the Micromeritcs Tristar 2420 analyzer (USA). The sample was vacuum degassed at 200 °C for 8 h before measurements. The specific surface area of the sample was calculated based on Brunauer-Emmett-Teller (BET) method, and the distribution of pore sizes was derived based on Barrett-Joyner-Halenda (BJH) model. The quantitative analysis of Ni^{2+} ions in electrolytes at different stages was determined by Inductive Coupled Plasma Emission Spectrometer (ICP) based on standard Ni^{2+} concentrations.

Synthesis of mesocrystal NiS_2 nanospheres (M-NiS_2)

In a typical procedure, 2.5 mmol of elemental sulfur powder was dispersed in 80 mL of TEG and then followed by the addition of 1.0 mmol of $Ni(NO_3)_2 \cdot 6H_2O$. Afterward, the mixture was magnetically stirred at room temperature (~25 °C) for 4 h until the metal salt was dissolved. Afterward, the solution was transferred into a 100 mL Teflon-lined autoclave and kept heating at 180 °C for 4 h. When the temperature was naturally cooled

to room temperature (~25 °C) after the reaction, the M-NiS_2 product was obtained after washing with water and ethanol, and drying at 60 °C for 12 h.

Electronic conductivity test

Based on the two-electrode method, the conductivity was tested by the EC-Lab electrochemical workstation. The calculation formula is as follows:[1,2]

$$\sigma = \frac{4HI}{V\pi D^2} \tag{1}$$

where *H* is the sheet thickness; *D* is the sheet diameter; *I* is the current; *V* is the voltage.

Calculation of ion diffusion coefficient

Hereafter, the copper-ion diffusion coefficient is calculated based on the following equation:[3]

$$D_{\mathrm{Cu}} = \frac{R^2T^2}{2A^2n^4F^4{C_{\mathrm{Cu}}}^2\sigma^2} \tag{2}$$

$$Z_{\mathrm{re}} = R_e + R_{\mathrm{ct}} + \sigma\omega^{-1/2} \tag{3}$$

where the value of the ideal gas constant R is 8.314 J mol^{-1} K^{-1}; the absolute Kelvin temperature T is 298.15 K; n represents the number of electrons transferred during the electrochemical reaction; F is the Faraday constant (96485 C mol^{-1}); A is the surface area of the cathode electrode; C_{Cu} is the concentration of copper ions in the electrolyte; and σ is the Warburg factor related to the Z_{re} parameter. As is calculated, the apparent ionic diffusion coefficients of the M-NiS_2 and C-NiS_2 electrodes were calculated to be 3.3×10^{-18} and 4.8×10^{-19} cm^2 s^{-1}, respectively, demonstrating the unique structure of the M-NiS_2 can facilitate ionic transportation.

Electrochemical Measurements

The M-NiS_2 electrode (working electrode) was prepared by mixing the 10:20:60:10 wt.% of carboxymethyl cellulose sodium (CMC-Na) binder, Ketjen black (KB), M-NiS_2 powder, and polymerized styrene butadiene rubber (SBR) binder, respectively. The above mixture slurry was cast on carbon cloth and then dried at 60°C in an oven for 12 h. The loading of M-NiS_2 was about 1-1.5 mg cm^{-2} and the diameter of the circular electrode was 1.2 cm. The preparation of the C-NiS_2 electrode was the same as above, and only M-NiS_2 powder was replaced by C-NiS_2.

The high-loading electrode (5-7 mg cm^{-2}) was fabricated by compressing a mixture of the M-NiS_2 powder, Ketjen black (KB), and poly(vinylidene difluoride) (PTFE, 5 wt% aqueous solution) binder in a mass ratio of 6:3:1 on the carbon cloth followed by drying at 60 °C for 12 h.

The electrode performance of the M-NiS_2 (or C-NiS_2) was tested on CR2032-type coin cells. The coin cells were assembled with M-NiS_2 as the working electrode, copper

mesh as the counter electrode, and glass fiber membranes (Whatman GF/D) were used as the separator. The $CuSO_4$ aqueous solution (0.5 mol L^{-1}) was used as the electrolyte in the battery. The operating voltage window of NiS_2-Cu cells was 0-0.36 V *vs.* Cu^{2+}/Cu. The galvanostatic charge-discharge (GCD) measurements were performed on the Neware battery test system (CT-4008T-5V50mA, Shenzhen, China). Cyclic voltammetry (CV) and electrochemical impedance spectroscopy (EIS) were operated on the EC-Lab electrochemical workstation (Biologic VSP-3e). For the EIS test, the frequency range from 10^{-1} to 10^5 Hz was used by applying a disturbance amplitude of 5 mV. In the *in-situ* EIS test, the discharge process was continued for 10 min at a current density of 0.5 A g^{-1} until the voltage reached 0 V (*vs.* Cu^{2+}/Cu) (or charging to 0.36 V *vs.* Cu^{2+}/Cu), and then the EIS test was performed after resting for 40 min. All of the above electrochemical performance tests were performed at room temperature (~ 25°C).

The theoretical capacity calculation for the six-electron reaction in our manuscript is based on the following reaction: 2S ↔ NiS_2 ($2e^-$) ↔ CuS + NiS ($2e^-$) ↔ Cu_2S + NiS ($2e^-$). And the theoretical capacity was calculated as 6 × 96486 ÷ (3.6 × 122.69) = 1311 mAh g^{-1} based on the initial NiS_2 material.

From the point of thermodynamics, the total Gibbs energy changes for the redox pairs of CuS/S and NiS_2/S were calculated, respectively. According to LANGE'S HANDBOOK OF CHEMISTRY (16 th), the Gibbs free energy values of Cu^+, Cu^{2+}, S, Cu_2S, CuS, NiS_2, and Ni^{2+} compounds are recorded as 50.0, 65.52, 0, −86.2, −53.7, −124.7, and −45.6 kJ mol^{-1}, respectively. The total Gibbs energy change for redox pairs of Cu_2S/S, CuS/S, and NiS_2/S was 186.2, 119.22, and 79.1 kJ mol^{-1}, respectively.

The full battery performance was detected by an H-type glass battery. To further improve the voltage window of the whole battery, the acid-base dual solution was used as the electrolyte. The bipolar membrane (Fumasep FBM-PK 130) was used to separate the catholyte (0.5 mol L^{-1} $CuSO_4$ + 1 mol L^{-1} H_2SO_4) and the anolyte (2 mol L^{-1} KOH + saturated ZnO), thus inhibiting the spontaneous neutralization. H_2SO_4 or other inert salts can be applied as additives for the catholyte to create an ionic equilibrium. The operating voltage window of NiS_2||Zn cells was set as 1.48-1.98 V.

Energy Density Calculation

The calculation of the half-cell energy density. At 1 A g^{-1}, the capacity of the NiS_2 is 1073 mAh g^{-1}. When considering the total mass of the discharged cathode that is NiS/Cu_2S/KB (considering that the mass ratio of NiS_2: KB is 3:1 during electrode fabrication), it would be 90.69 + 160 + 40.89 = 291.58 g. Therefore, the specific capacity of the NiS/Cu_2S/KB composite is 1073 mAh g^{-1}× 122.69 g / 291.58 g = 451.48 mAh g^{-1}. Considering the average operation voltage of the hybrid cell of 1.6 V, the half-cell energy density is 451.48 mAh g^{-1} × 1.6 V = 722.4 Wh kg^{-1}. And this hybrid cell delivers specific capacity and energy of 270.5 mAh g^{-1} and 432.9 Wh kg^{-1}, respectively, based on active materials of both electrodes (NiS/Cu_2S/KB and Zn)

The theoretical energy density of the full cell was calculated as follows: 1 mol of NiS_2, 2 mol of $CuSO_4$ salt, and 3 mol of Zn metal were needed based on the six-electron reaction. In total, the total material's mass of both electrodes (considering that the mass ratio of NiS_2: KB is 3:1 during electrode fabrication) was 122.69+ 40.89 + 320 + 3 ×

65 = 678.58 g. At 1 A g^{-1}, the theoretical capacity for the full cell can be calculated as 1311 mAh g^{-1} × 122.69 g/ 678.59 g = 237.03 mAh g^{-1}. Hence, the theoretical energy density was estimated as 237.03 mAh g^{-1} × 1.6 V = 379.2 Wh kg^{-1}. For practical applications, we need to consider the real capacity of the M-NiS_2, the plating efficiency of zinc metal, and the mass of the bipolar membrane and electrolyte.

Cost Estimation

Cost estimation for electrode materials and NiS_2||Zn cell device. The estimated combined cost for the cathode (based on NiS/Cu_2S/KB) and anode active material can be calculated as: $(m_A \times P_A + m_C \times P_C)/E_a$ = [(0.195 × 3.5) + (0.12269 × 76.5) + (0.04089 × 5.6)]/[0.4329 × (0.195 + 0.12269 + 0.04089 + 0.128)] = 48.89 US$ kWh^{-1}, where P_A and P_C correspond to the price of anode and cathode in US$ kg^{-1}, respectively; and E_a is the actual energy in kWh. As shown in the calculation results of the previous section, the actual energy density of 432.9 Wh kg^{-1} can be reached at 1A g^{-1}, and the materials cost of the NiS_2||Zn cell is USD $48.89 per kWh. Bipolar membrane for cell assembly costs around 2.8 US$ (2 × 3 cm). The cost of the aqueous electrolyte is almost negligible when compared to the cost of the above materials. Additional costs would arise slightly from cell components and housing, which are not taken into account. Therefore, the total cost of the NiS_2||Zn cell is around USD $51.69 per kWh.

Material	Price	Information source
NiS_2	76.5 US$ kg^{-1}	b2b.baidu.com
Zn foil	3.5 US$ kg^{-1}	b2b.baidu.com
KOH	0.5 US$ kg^{-1}	b2b.baidu.com
$CuSO_4$	1.2 US$ kg^{-1}	b2b.baidu.com
H_2SO_4	0.06 US$ kg^{-1}	Tonghuashun iFinD (November 4, 2022)
Ketjen black (KB)	5.6 US$ kg^{-1}	Alibaba.com
Bipolar membrane (Fumasep FBM-PK 130)	4592.9 US$ m^{-2}	Taobao.com

Calculation of electrode activation energy

The activation energy was calculated by the Arrhenius formula:

$$\ln k = \frac{-E_a}{RT} + \ln A \tag{4}$$

$$E_{\mathrm{a}} = \frac{|\mathrm{K}| \times 1000 \times R}{\mathrm{NA} \times 1.6 \times 10^{-19}} \tag{5}$$

Here, the value of the ideal gas constant R is 8.314 J mol^{-1} K^{-1}; k is represented by $R_{ct}{}^{-1}$ (Ω^{-1} cm^{-2}); the slope K is obtained from the $-\ln(R_{ct}{}^{-1})$-T^{-1} image; and NA is Avogadro constant.

DRT fitting based on impedance data

DRT was calculated from impedance data by using open-source Python scripting software (DRT Tools). The Tikhonov regularization method was used to fit the discrete experimental data, and the data was discretized based on the Gaussian method. On the premise of eliminating the inductive reactance part, the real and imaginary parts of the EIS were fitted at the same time. The second-order regularization derivative fitting parameter was chosen, the regularization parameter was set to 0.0001 (the difference between the residual actual impedance and the impedance of the DRT was in the acceptably low range), and the basis function (RBF) fitting using a radial FWHM of 0.5 was used [4].

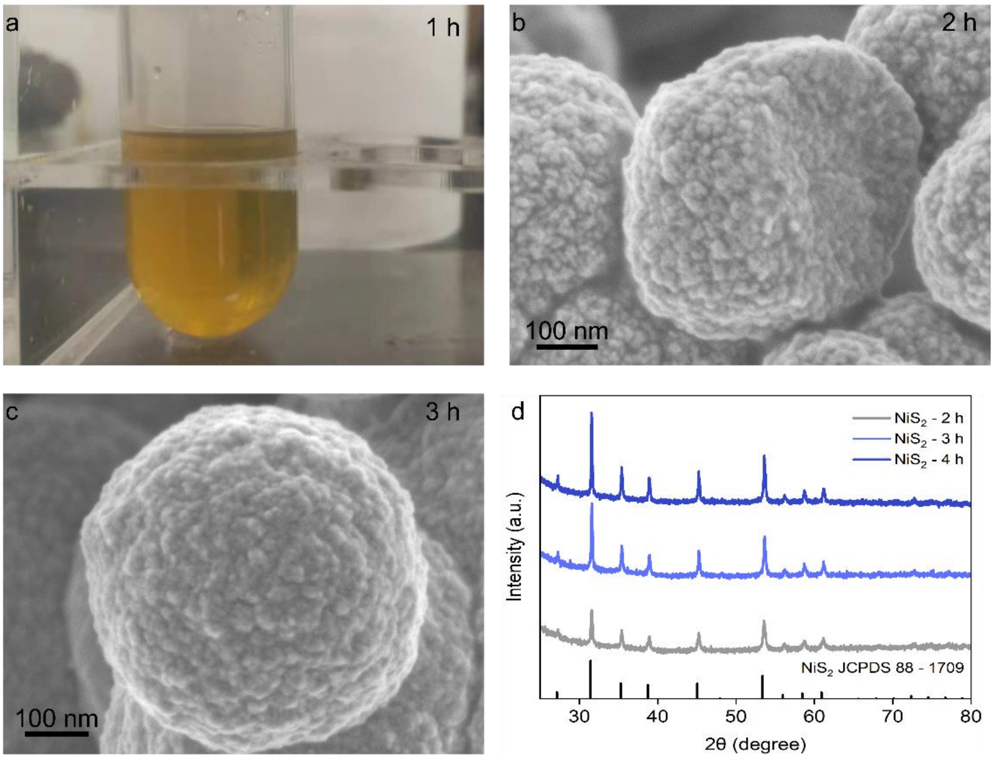

Supplementary Figure 1. The photograph, morphologies, and structure of the M-NiS_2 at different reaction times. (a) Photograph of the M-NiS_2 after reacting 1 h (homogeneous solution, no precipitated product after centrifugation). SEM images of the M-NiS_2 after reacting (b) 2h and (c) 3h. (d) XRD patterns of the M-NiS_2 at each reaction time. The above results indicate that the nucleation of the NiS_2 did not occur at the initial stage of the reaction. With the further progress of the reaction, the strength of the (200) crystal plane increased the most, and the oriented growth behavior of the M-NiS_2 material was further confirmed.

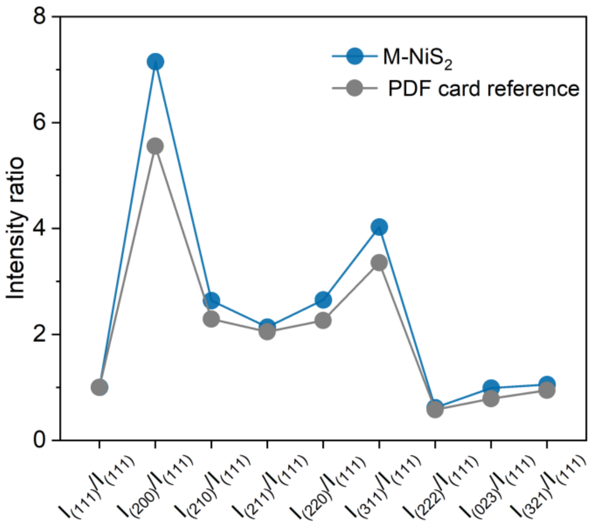

Supplementary Figure 2. Comparison of peak intensity ratios for the experimental and reference data.

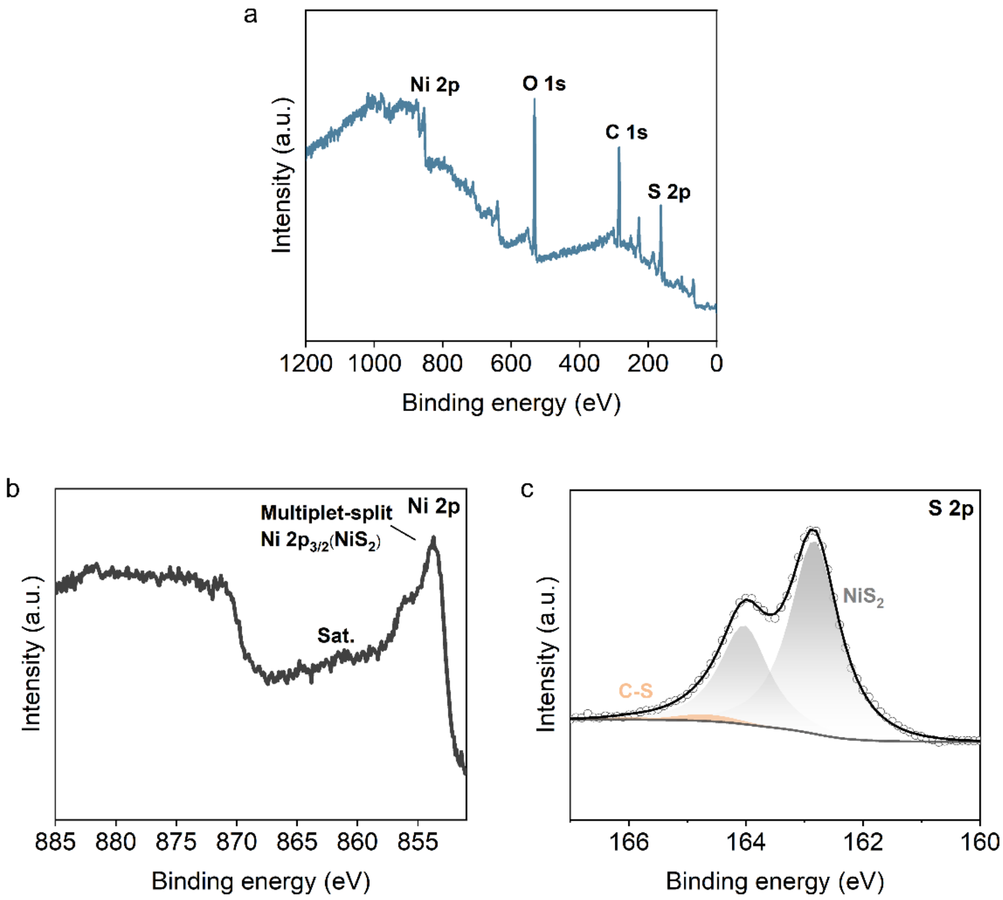

Supplementary Figure 3. XPS characterization of the M-NiS$_2$. (a) Survey spectrum. (b) Ni 2p spectrum. (c) S 2p spectrum.

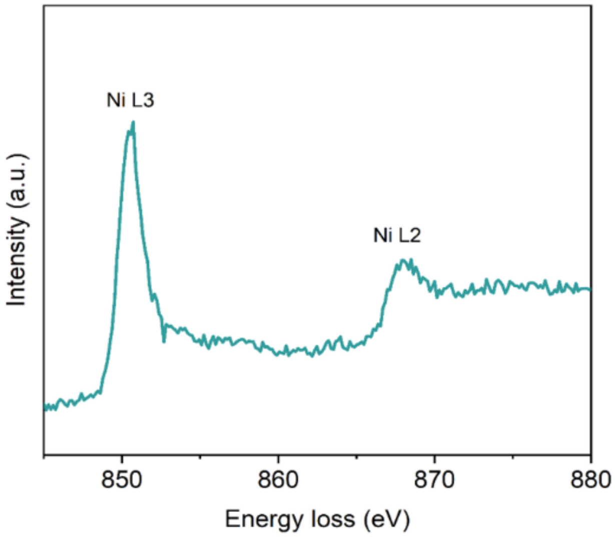

Supplementary Figure 4. Background-subtracted EELS of Ni $L_{2,3}$ edge obtained from the M-NiS_2 sample.

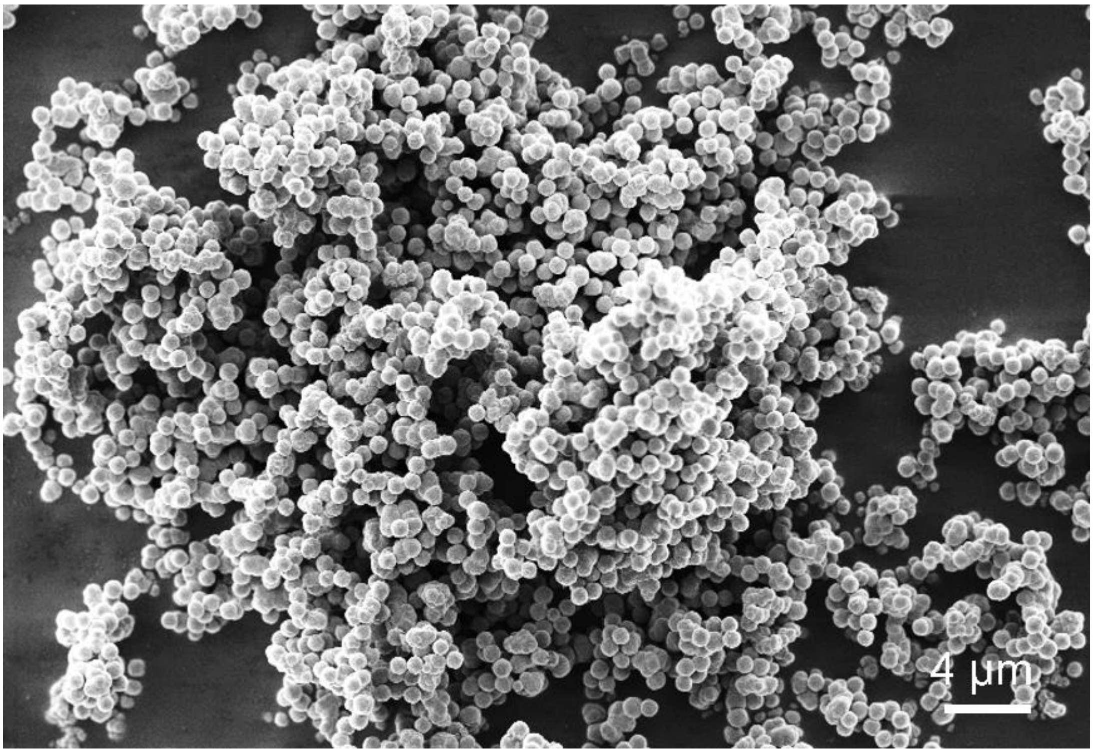

Supplementary Figure 5. Low-magnification SEM image of the M-NiS_2.

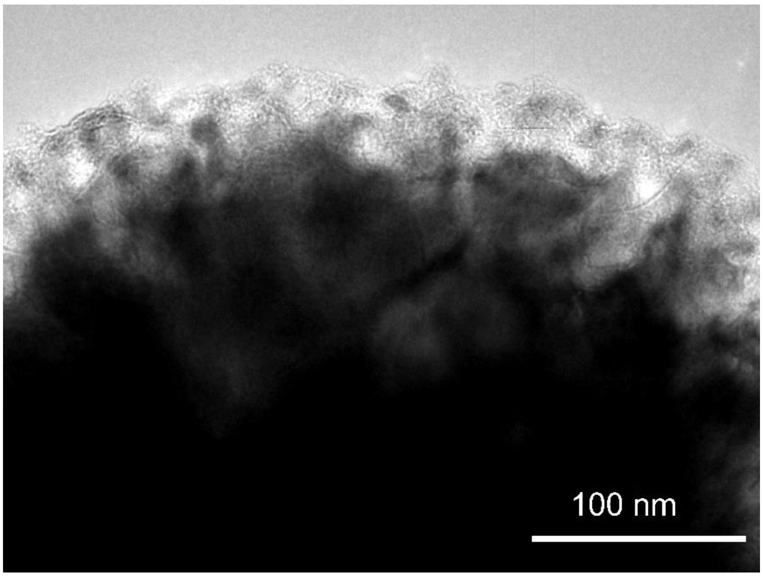

Supplementary Figure 6. TEM image of the M-NiS_2 single nanosphere.

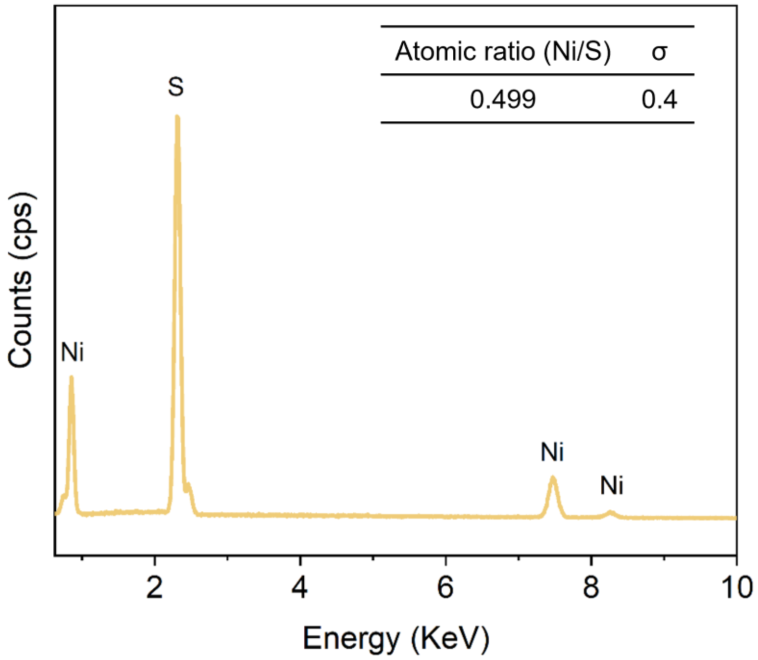

Supplementary Figure 7. EDX spectrum of the M-NiS_2.

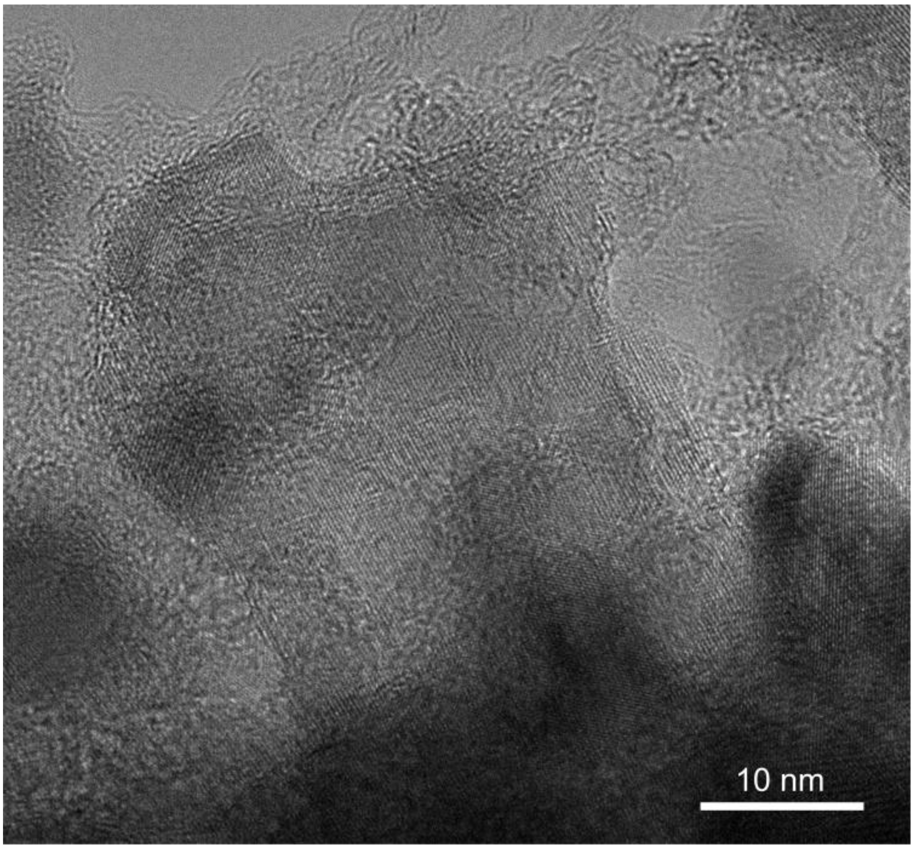

Supplementary Figure 8. The high-angle annular dark-field scanning transmission electron microscopy (HAADF-STEM) image of the M-NiS_2.

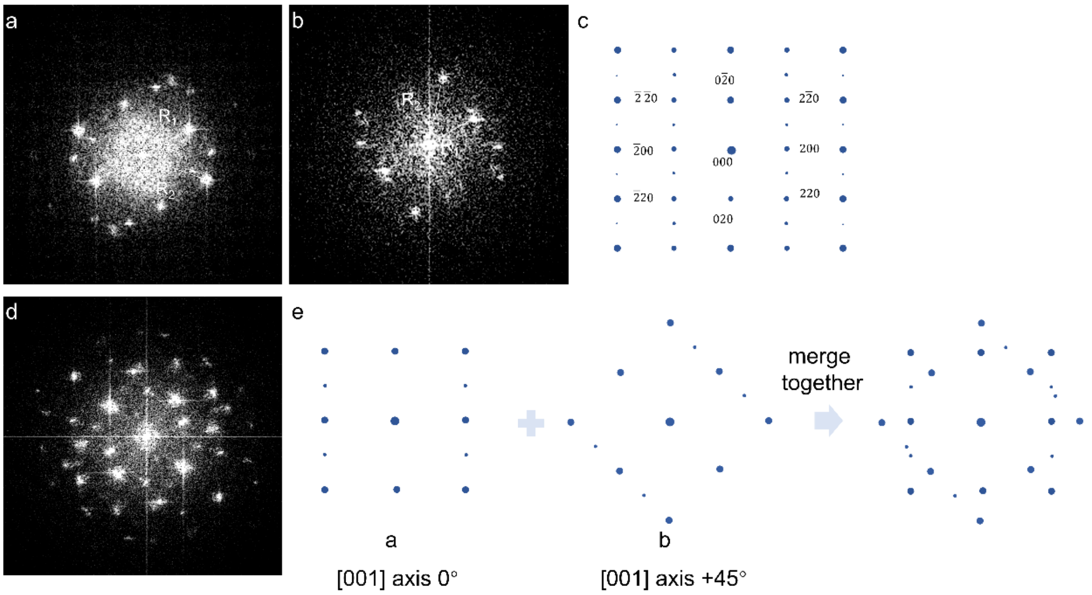

Supplementary Figure 9. FFT diffraction patterns and corresponding simulated electron diffraction pattern of the M-NiS_2. (a) FFT plot of the I region in Figure 1h. (b) FFT plot of the II region in Figure 1h ([001] zone axis). (c) Simulation [001] electron diffraction spectrum. (d) FFT diffraction pattern of the whole sample. (e) Superposition of simulated [001] electron diffraction spectra of different grains.

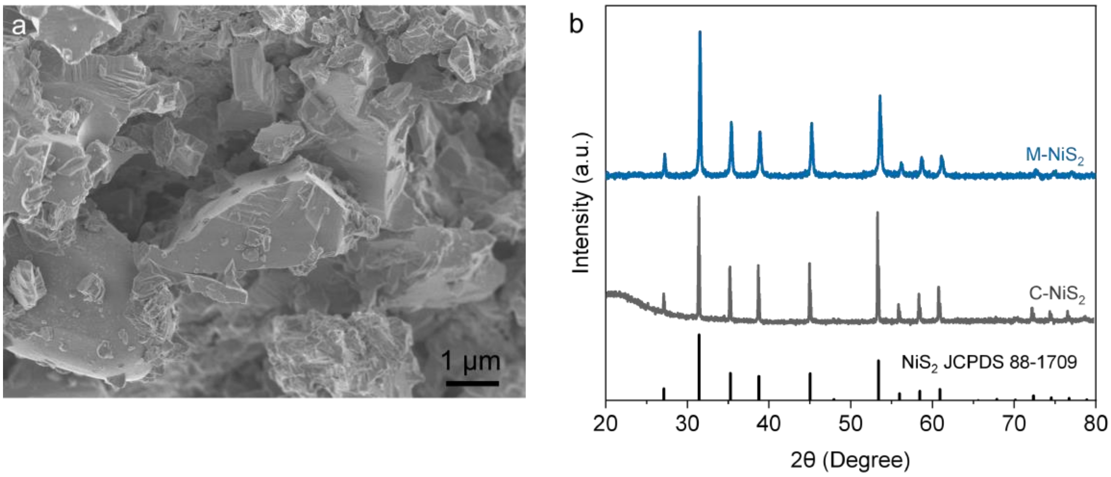

Supplementary Figure 10. (a) SEM image of the C-NiS_2. (b) The phase comparison of C-NiS_2 and M-NiS_2.

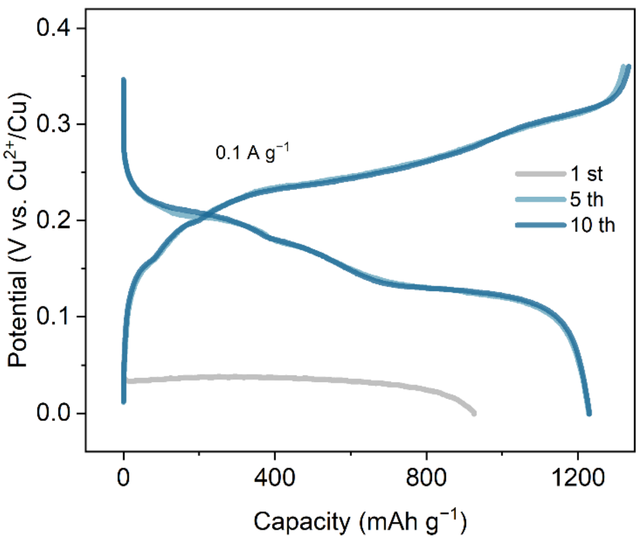

Supplementary Figure 11. The activation process of the M-NiS_2 electrode at a current density of 0.1 A g^{-1}.

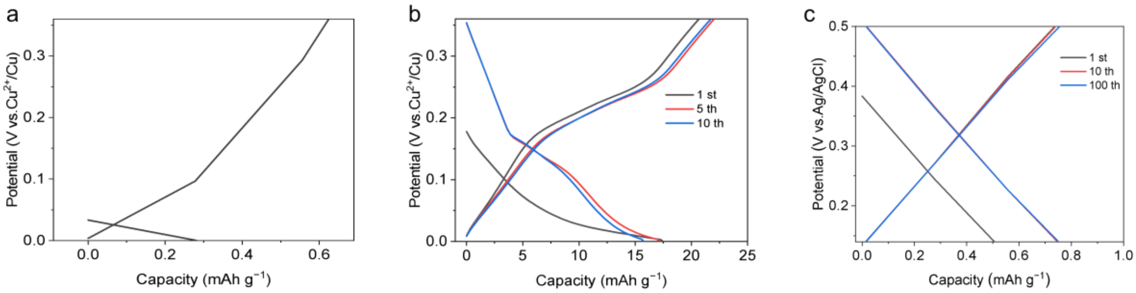

Supplementary Figure 12. (a) Discharge/charge curves of commercial carbon cloth. (b) Discharge/charge curves of electrode slurry. (c) Discharge/charge curve of M-NiS_2 in 1 mol L^{-1} H_2SO_4. The above results indicate that the additional capacity contribution from carbon cloth, slurry ingredients, and H^+ ions is negligible.

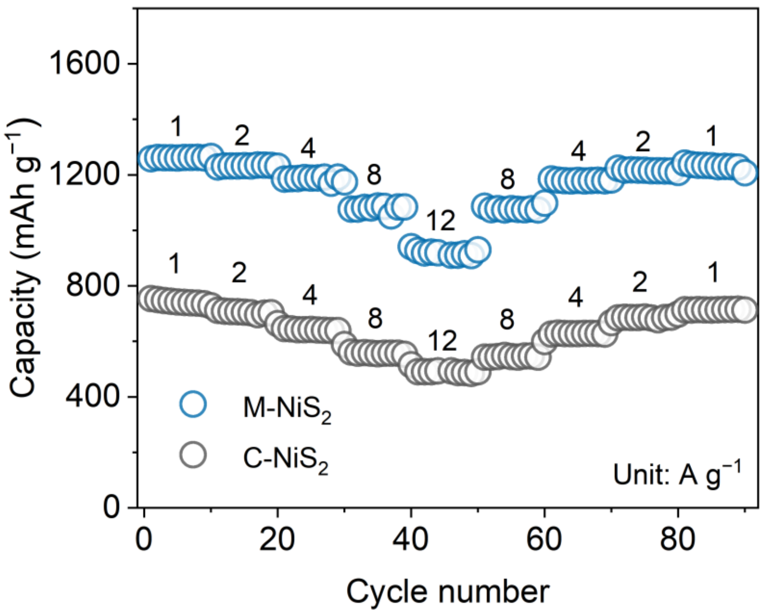

Supplementary Figure 13. Rate cycling performance of the M-NiS_2-Cu cell and the C-NiS_2-Cu cell under the discharge condition.

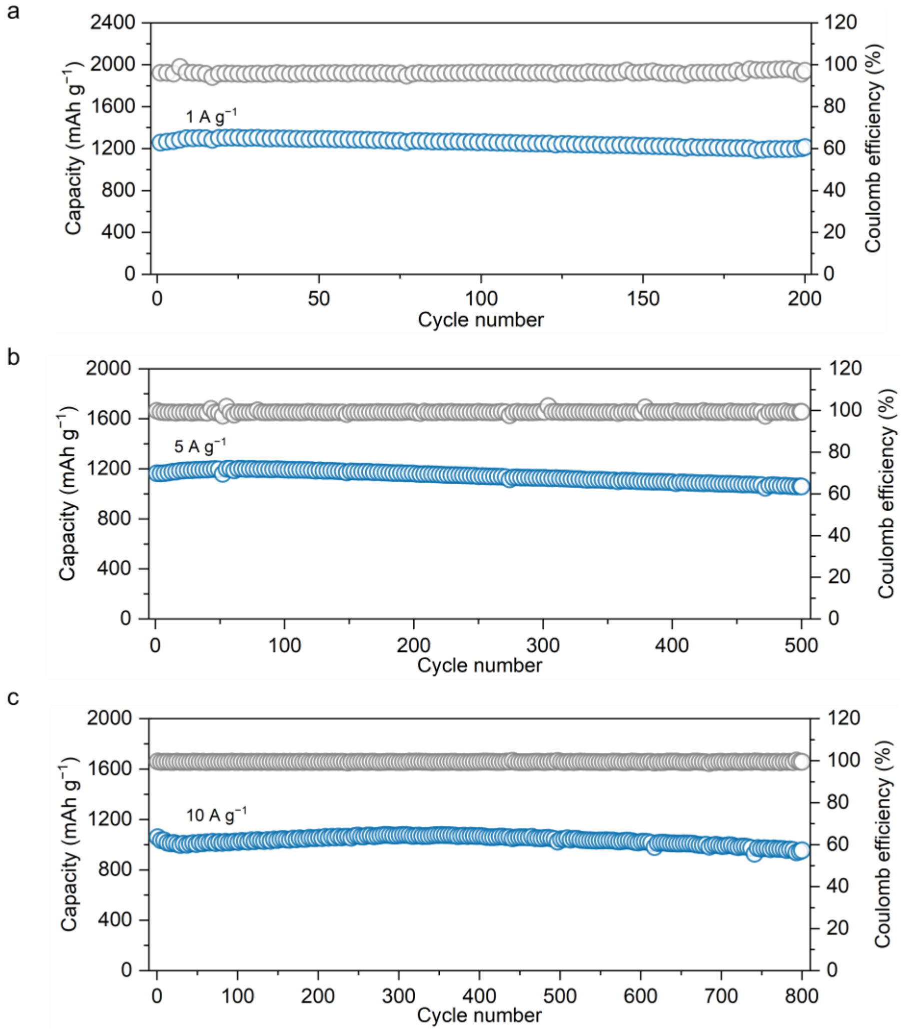

Supplementary Figure 14. Long cycle test of the M-NiS_2-Cu cell at (a) 1, (b) 5, and (c) 10 A g^{-1}.

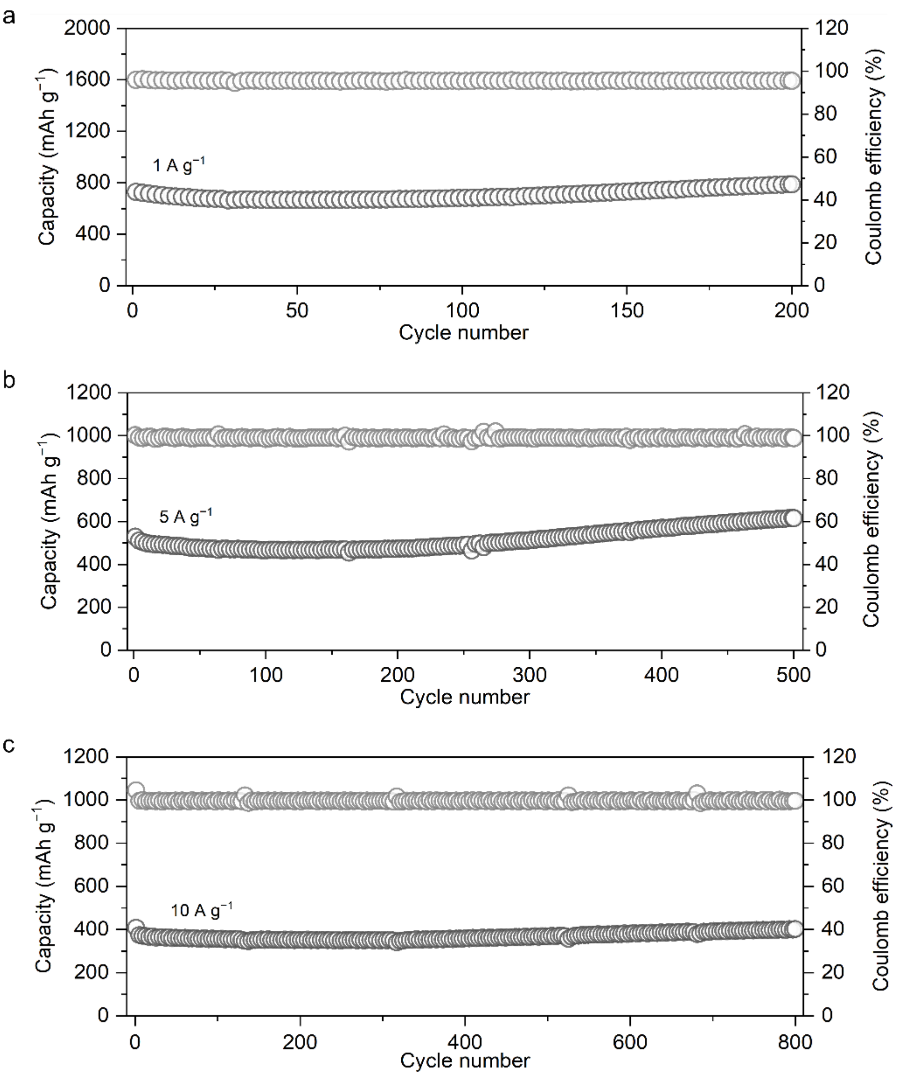

Supplementary Figure 15. Long cycle test of the C-NiS_2-Cu cell at (a) 1, (b) 5, and (c) 10 A g^{-1}.

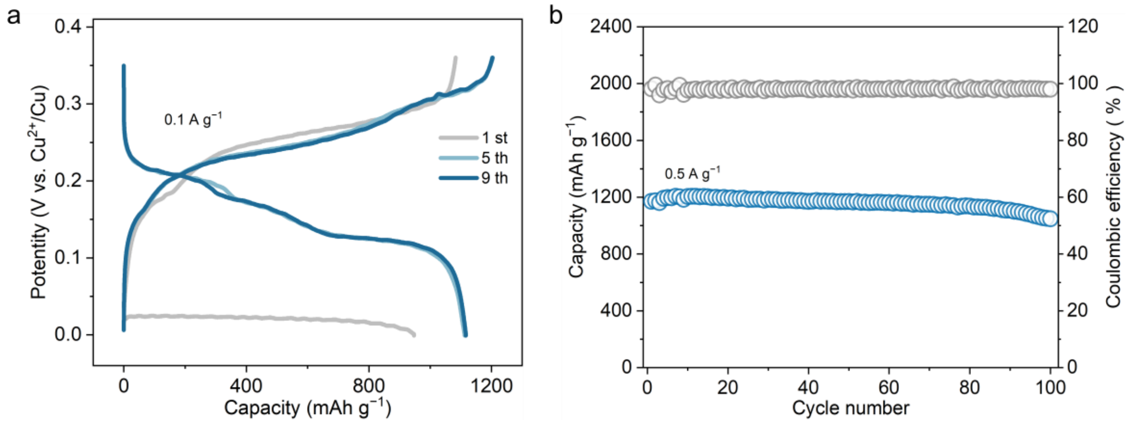

Supplementary Figure 16. (a) GCD profile and (b) cycle stability of the M-NiS_2 electrode with a high mass load of 5-7 mg cm^{-2}.

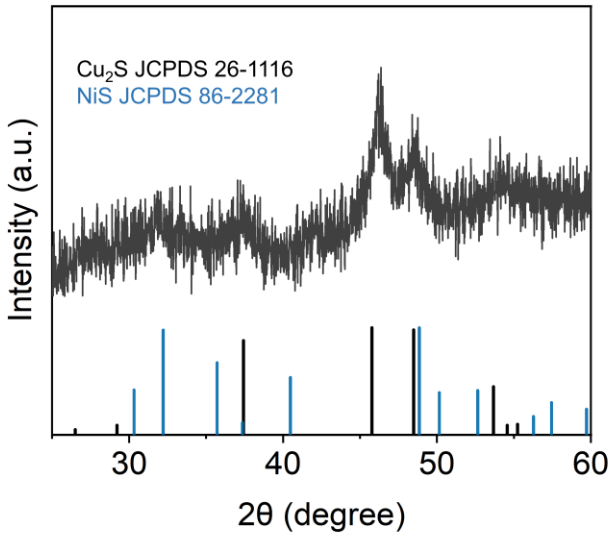

Supplementary Figure 17. The XRD pattern of the M-NiS_2 electrode after initial discharge.

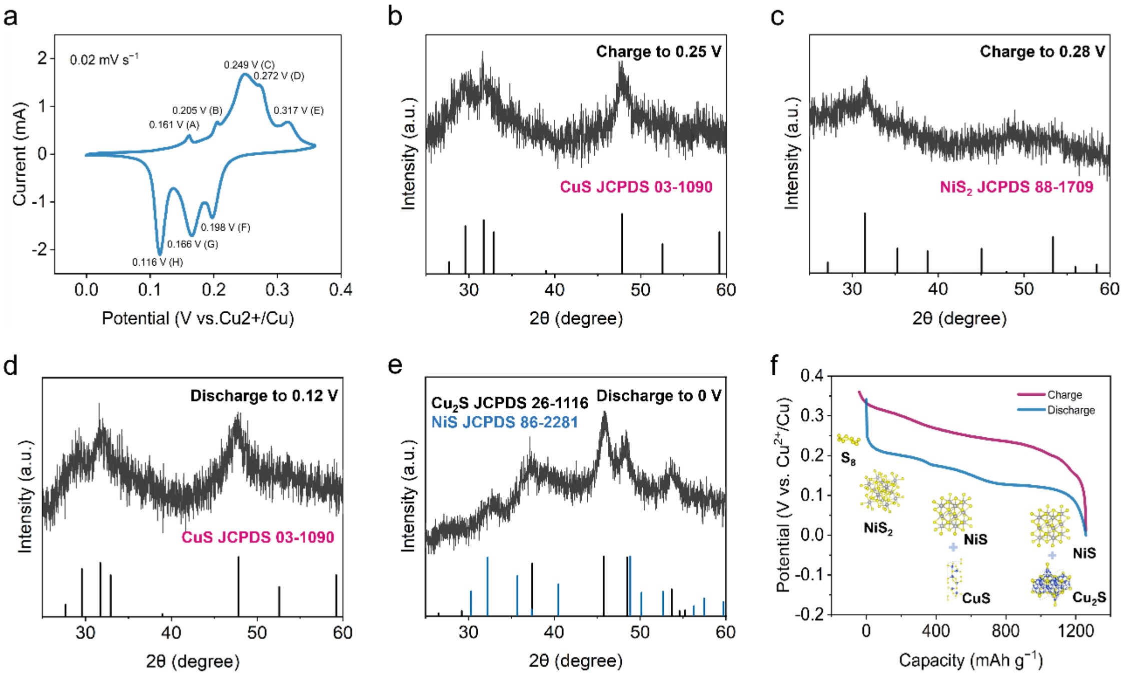

Supplementary Figure 18. The phase transition process of the M-NiS$_2$ electrode at different stages. (a) CV curve at a scan rate of 0.02 mV s^{-1}. The *ex-situ* XRD patterns of the M-NiS$_2$ electrode after (b) charge to 0.25 V, (c) charge to 0.28 V, (d) discharge to 0.12 V, and (e) discharge to 0 V. (f) Phase transitions of the M-NiS$_2$ electrode at different states.

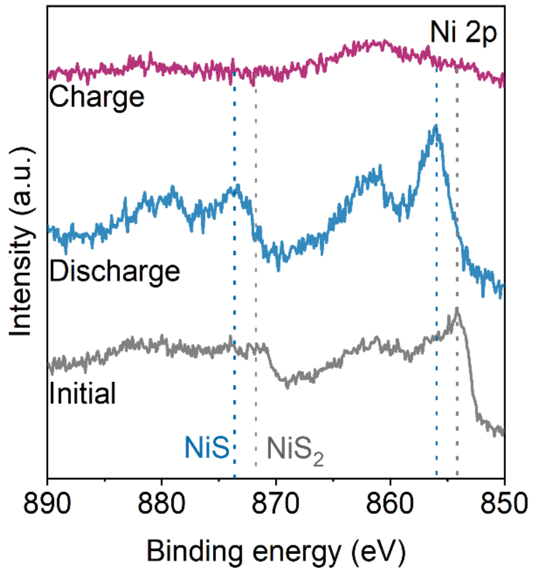

Supplementary Figure 19. Ni 2p spectra of M-NiS$_2$ electrode at different charge/discharge stages.

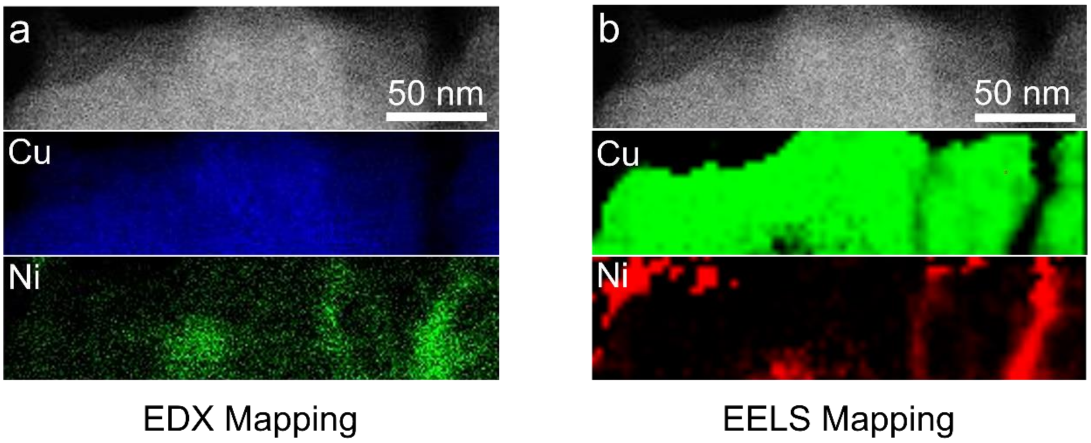

Supplementary Figure 20. The (a) EDX mapping and (b) ELLS mapping of the M-NiS_2 after discharge.

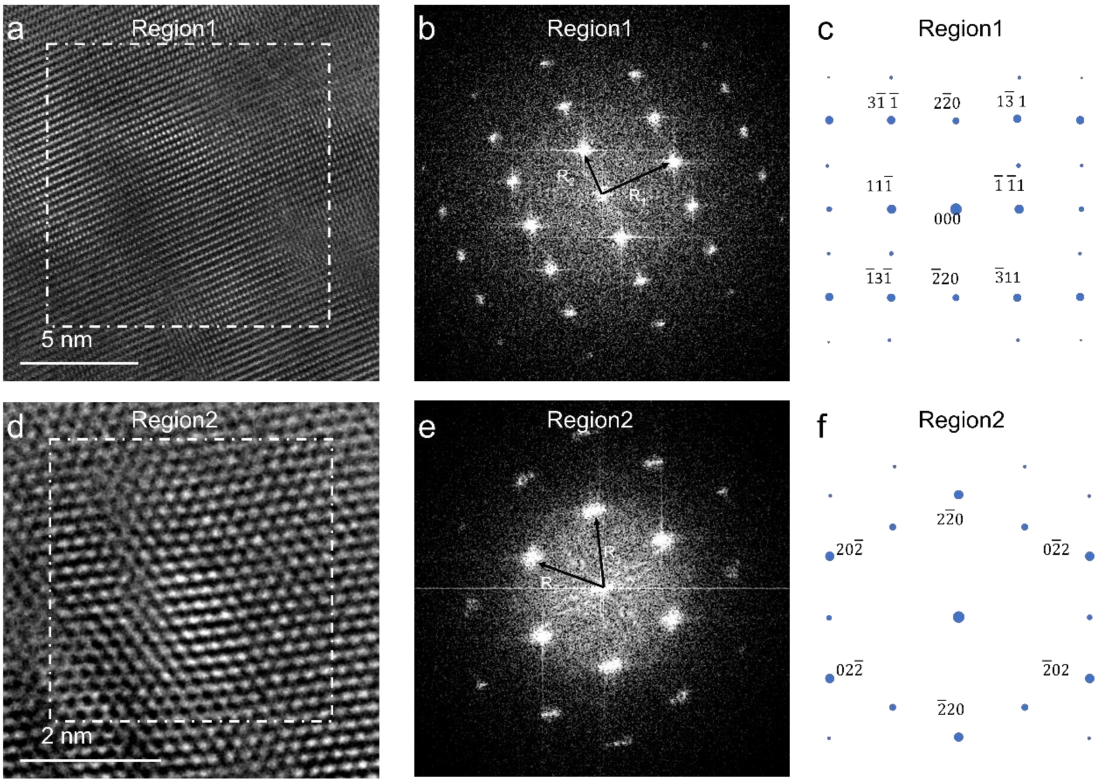

Supplementary Figure 21. HRTEM, corresponding FFT, and simulated images of the M-NiS_2 sample after charging at different regions. (a) HRTEM image. (b) FFT image ([112] zone axis). (c) Simulation electron diffraction pattern of [112] zone axis. (d) HRTEM image. (e) The corresponding FFT image ([111] zone axis). (f) Simulation electron diffraction pattern of [111] zone axis.

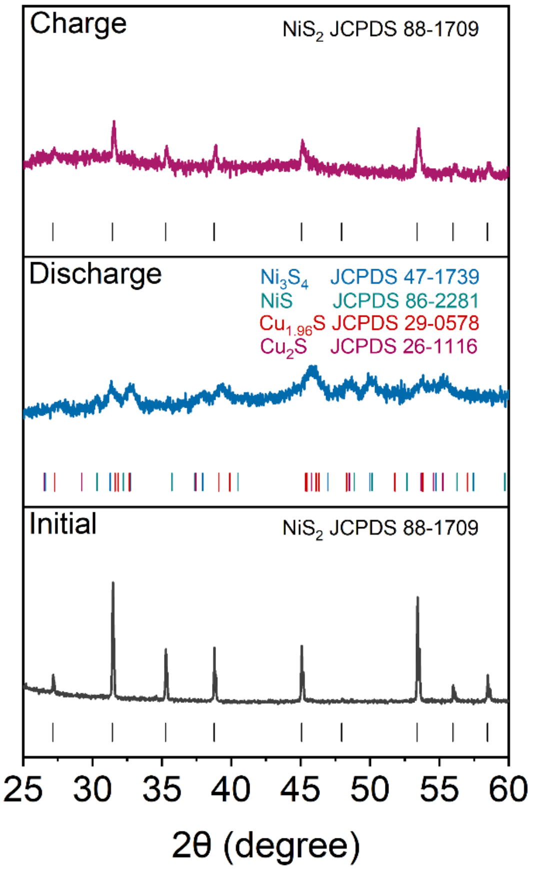

Supplementary Figure 22. *Ex-situ* XRD patterns of C-NiS_2 electrode in different states. (In addition to the products Cu_2S and NiS, the final discharge state of the C-NiS_2 electrode also has $Cu_{1.96}S$ and Ni_3S_4 intermediate phases. And the characteristic peak of NiS_2 after charging is more obvious than that of the M-NiS_2 electrode, indicating that the charging product is NiS_2. The above results confirm that the M-NiS_2 electrode has better electrochemical reaction kinetics than the C-NiS_2 electrode.)

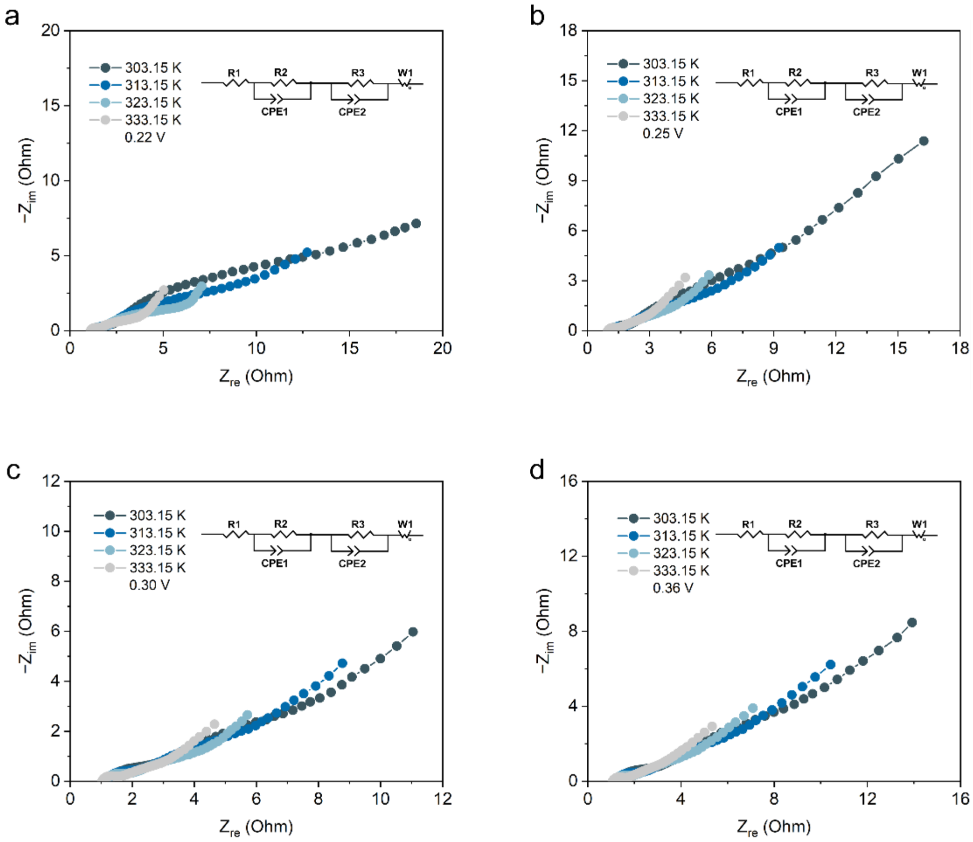

Supplementary Figure 23. EIS curves of the M-NiS_2 electrode at various voltages and temperatures.

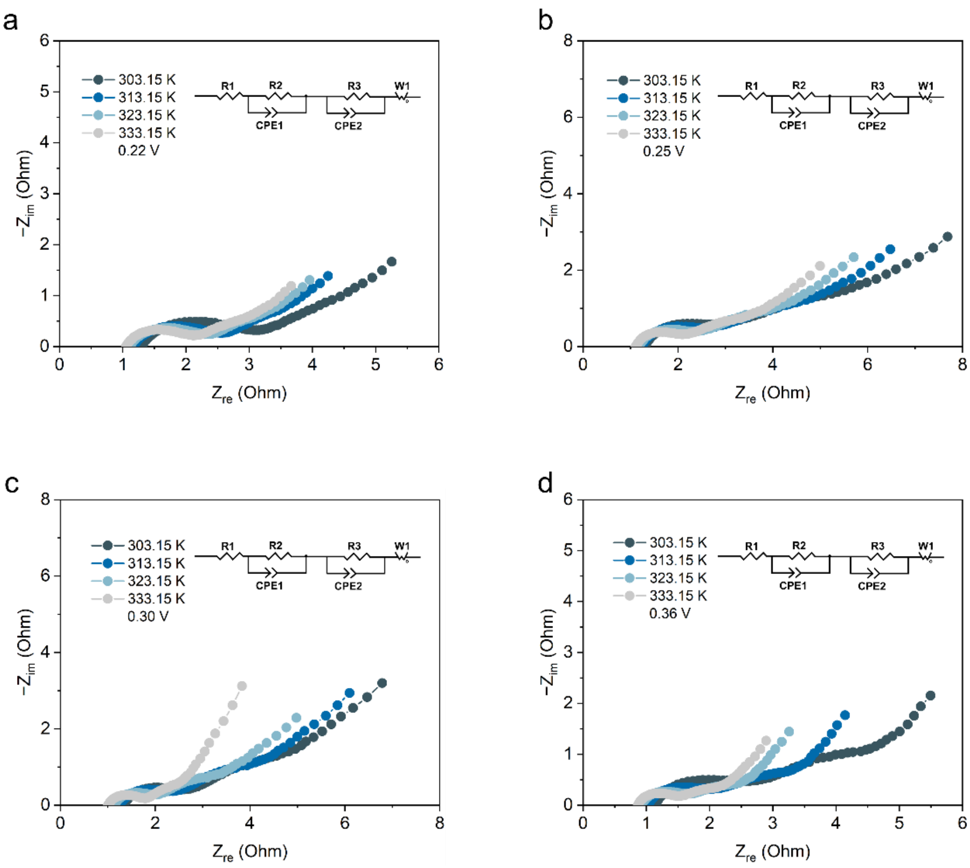

Supplementary Figure 24. EIS curves of the C-NiS_2 electrode at various voltages and temperatures.

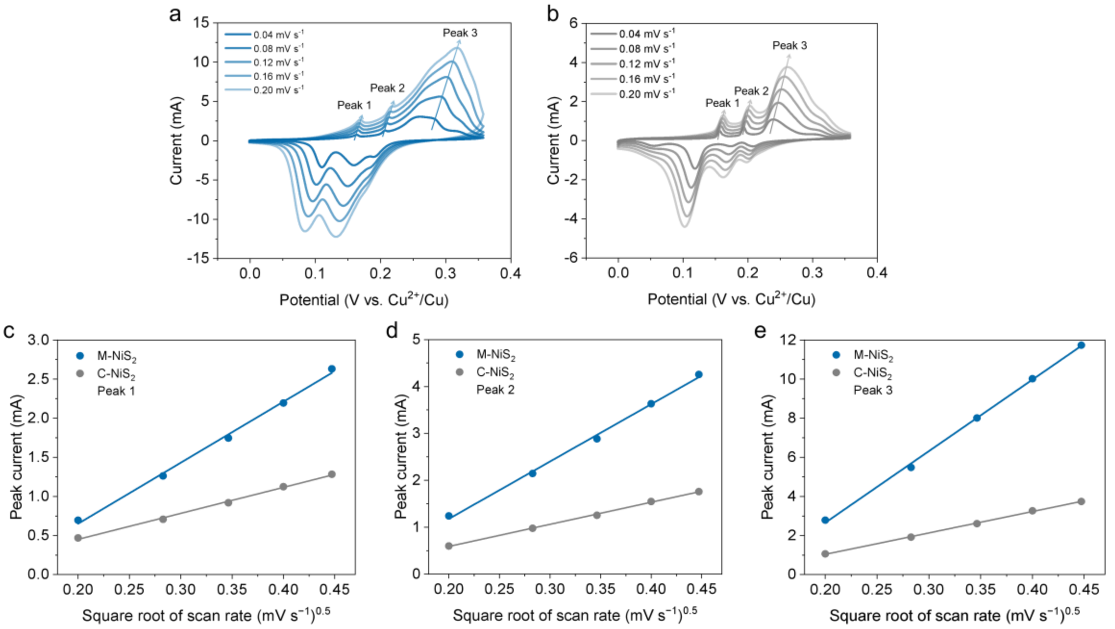

Supplementary Figure 25. CV curves of (a) M-NiS_2 and (b) C-NiS_2 at different scan rates. (c) The first anodic oxidation process (Peak 1) *vs.* the square root of the scan rates. (d) The second anodic oxidation process (Peak 2) *vs.* the square root of the scan rates. (e) The third anodic oxidation process (Peak 3) *vs.* the square root of the scan rates.

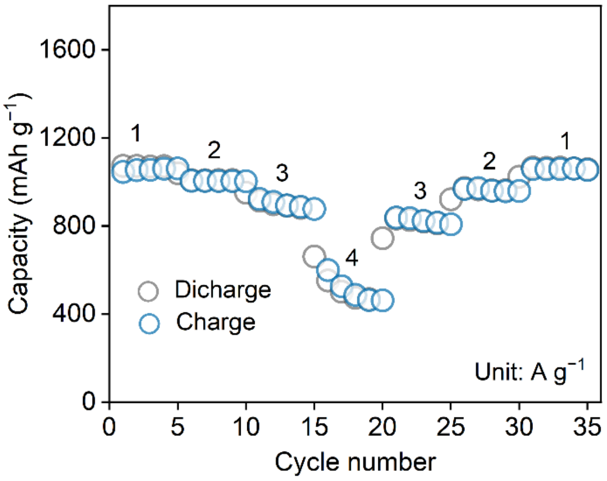

Supplementary Figure 26. The rate performance of the hybrid M-NiS_2||Zn full cell.

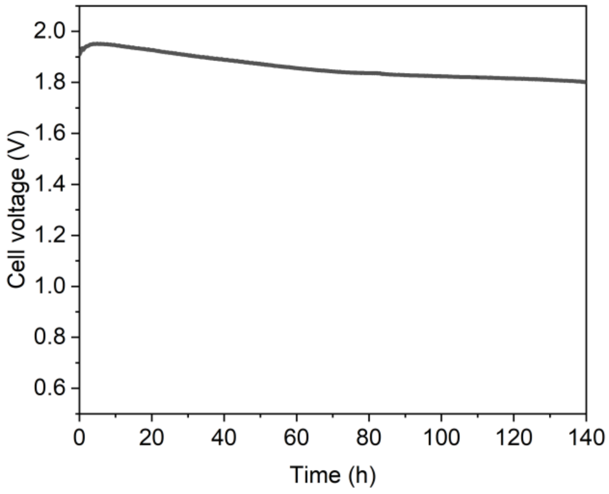

Supplementary Figure 27. The self-discharge standing test of the M-NiS_2||Zn full cell.

Supplementary

Table 1 Electronic conductivity values of the M-NiS_2 and C-NiS_2 powder.

Samples	Electrical conductivity (S cm^{-1})
M-NiS_2	15.53
C-NiS_2	0.17

Supplementary Table 2 The ICP values of the Ni ion concentration in the electrolyte at different stages of the M-NiS_2-Cu cell.

Sample (Ni^{2+})	1 (μg ml^{-1})	2 (μg ml^{-1})	3 (μg ml^{-1})	Average (μg ml^{-1})
1st Discharge	0	0	0	0
1st Charge	1.37	1.37	1.43	1.39
2nd Discharge	0.08	0.08	0.10	0.09

The numbers of "1", "2", and "3" represent the number of the three parallel samples. The loading of the NiS_2 electrode is about 2 mg with an electrolyte amount of 160 μl. The electrolyte is then diluted to 4000 times the original value for ICP testing. The ICP test results show that no Ni ions can be detected in the electrolyte after the first discharge, indicating that Ni exists on the electrode in the form of solid NiS in the first SRR process. After the first charging, a large amount of Ni ions can be detected in the electrolyte, indicating that Ni ions are released into the electrolyte in the first SOR process. After the 2nd discharging, neglectable Ni ions can be detected in the solution, indicating the updates of Ni^{2+} in the 2nd SRR process, and the Ni exists in the electrode in the form of a solid state.

Supplementary Table 3 The fitted transfer resistance values for the M-NiS_2 and C-NiS_2 electrodes at varying voltages and temperatures.

		0.22 V	0.25 V	0.30 V	0.36 V
M-NiS_2	303 K	22.14	20.70	28.95	51.04
M-NiS_2	313 K	14.24	15.92	16.25	30.68
M-NiS_2	323 K	8.29	12.75	9.03	20.47
M-NiS_2	333 K	4.95	9.44	5.18	13.37
C-NiS_2	303 K	77.84	12.40	43.40	11.74
C-NiS_2	313 K	24.06	9.31	17.29	6.09
C-NiS_2	323 K	6.85	7.04	7.97	3.56
C-NiS_2	333 K	2.91	6.05	3.67	2.25

(the value of the R_{ct} cm^{-2} is the transfer resistance value fitted in the table divided by the surface area of the electrode)

Supplementary Table 4. Comparison of electrochemical properties of various aqueous systems.

System	Discharge voltage (V)	Capacity[a)] (mAh g^{-1}/ A g^{-1})	Half-cell Energy density [b)] (Wh kg^{-1})	Retention /Cycles
Zn||S[5]	0.58	332.1/0.5	192.6	54.0%/200
Fe||S[6]	0.38	230.1/0.2	87.4	66.7%150
S||$Ca_{0.44}MnO_2$[7]	1.29	149.3/0.1675	192.6	83.0%/150
S||$Na_{0.44}MnO_2$[8]	1.2	247.4/0.8375	296.9	82.0%/300
Al||S[9]	0.9	337.7/0.05	303.9	78.0%/300
Cu-S||Zn[10]	1.15	462.4/1	531.8	88.0%/110
Pb-S||Zn[11]	0.75	160.9/0.5	120.7	/
CuS||Zn[12]	1.22	306/2	373.3	90.0%/250
M-NiS_2||Zn	1.60	451.5/1	722.4	89.6%/250

[a)](the electrode capacity calculation is based on the total mass of active material (the material mass of the cathode electrode is the C/M_xS_y composite)); [b)](the calculation of the half-cell energy density is based on the material's mass of the C/M_xS_y composite)

Supplementary References

1. Li H, Guo C and Zhang T *et al.* Hierarchical confinement effect with zincophilic and spatial traps stabilized Zn-based aqueous battery. *Nano Lett* 2022, **22**, 4223-31.
2. Liang J, Tong K and Pei Q. A water-based silver-nanowire screen-print ink for the fabrication of stretchable conductors and wearable thin-film transistors. *Adv Mater* 2016, **28**, 5986-96.
3. Wang L, Zhao J and He X *et al.* Electrochemical Impedance Spectroscopy (EIS) study of $LiNi_{1/3}Co_{1/3}Mn_{1/3}O_2$ for Li-ion batteries. *Int J Electrochem Sc*. 2012; **7**: 345-53.
4. Soni R, Robinson J B and Shearing P R *et al.* Lithium-sulfur battery diagnostics through distribution of relaxation times analysis. *Energy Storage Mater* 2022; **51**: 97-107.
5. Zhang H, Shang Z and Luo G *et al.* Redox catalysis promoted activation of sulfur redox chemistry for energy-dense flexible solid-state Zn-S battery. *ACS Nano* 2021; **16**: 7344-51.
6. Wu X, Markir A and Xu Y *et al.* Rechargeable iron-sulfur battery without polysulfide shuttling. *Adv Energy Mater* 2019; **9**: 1902422.
7. Tang X, Zhou D and Zhang B *et al.* A universal strategy towards high-energy aqueous multivalent-ion batteries. *Nat Commun* 2021; **12**: 2857-67.
8. Kumar M and Nagaiah T C. High energy density aqueous rechargeable sodium-ion/sulfur batteries in ‘water in salt” electrolyte. *Energy Storage Mater* 2022; **49**: 390-400.
9. Huang Z, Wang W and Song W L *et al.* Electrocatalysis for continuous multi-step reactions in quasi-solid-state electrolytes towards high-energy and long-life Aluminum-sulfur batteries. *Angew Chem Int Ed Engl* 2022; **61**: e202202696.
10. Wu X, Markir A and Ma L *et al.* A four-electron sulfur electrode hosting a Cu^{2+}/Cu^{+} redox charge carrier. *Angew Chem Int Ed Engl* 2019; **58**: 12640-5.
11. Xu C, Yang Z and Yan H *et al.* Synergistic dual conversion reactions assisting Pb-S electrochemistry for energy storage. *Proc Natl Acad Sci USA* 2022; **119**: e2118675119.
12. Wang Y, Chao D and Wang Z *et al.* An energetic CuS-Cu battery system based on CuS nanosheet arrays. *ACS Nano* 2021; **15**: 5420-7.
